# Huajuhong: Insights into Botany, Phytochemistry, Pharmacology, and Applications in Food, Pharmaceuticals and Beyond

**DOI:** 10.3390/molecules31213918

**Published:** 2026-10-30

**Authors:** Jin Lu, Anlu Liao, Junyi Wang, Zhili Gao, Kunze Du, Jin Li, Xiaoyan Wang, Yanxu Chang

**Affiliations:** 1 State Key Laboratory of Chinese Medicine Modernization, Tianjin University of Traditional Chinese Medicine, Tianjin 301617, China; 15187059418@163.com (J.L.); 15632362106@163.com (A.L.); wjy081012@126.com (J.W.); giles_07@163.com (Z.G.); dkztcm@tjutcm.edu.cn (K.D.); lijin@tjutcm.edu.cn (J.L.); 2 Haihe Laboratory of Modern Chinese Medicine, Tianjin 301617, China

**Keywords:** *Exocarpium Citri Grandis*, flavonoids, ethnopharmacology, phytochemistry, pharmacology, applications

## Abstract

Huajuhong (*Exocarpium Citri Grandis*, ECG) is derived from *Citrus grandis* “Tomentosa” or *Citrus grandis* (L.) Osbeck and has long been used for respiratory health care. Growing interest in natural functional ingredients has drawn increasing attention to ECG as a multifunctional raw material. This review summarizes the botany, ethnopharmacology, phytochemistry, biological activities, and applications of ECG, with particular attention to its amino acid and mineral profiles. A total of 76 flavonoids reported from ECG were compiled. Flavones and flavanones predominated, and naringin was the major constituent. The available in vitro and in vivo studies indicate that ECG and its constituents exhibit antitussive, expectorant, hypoglycemic, hypolipidemic, anti-inflammatory, and antioxidant effects. The underlying mechanisms mainly involve the modulation of smooth-muscle function, the regulation of digestive enzyme activity and lipid-metabolic signaling, maintenance of iron homeostasis, inhibition of inflammatory mediator release, and enhancement of endogenous antioxidant defenses. These properties support its application in teas, functional beverages, lozenges, oral formulations, and other health-oriented products. However, its development is hampered by raw material variability, imperfect quality criteria, limited clinical evidence in humans, and insufficient understanding of ADME and oral bioavailability. Further research on standardized grading systems, constituent–function relationships, and clinical validation is required to support the rational use of ECG.

## 1. Introduction

*Citrus grandis* belongs to the Rutaceae family. It is a species native to northeastern India, northern Myanmar, and northwestern Yunnan [1]. It has been cultivated in China for over 3000 years. The dried outer pericarp of its immature or nearly mature fruits is used in traditional Chinese medicine (TCM) as *Exocarpium Citri Grandis* (ECG), also known as Huajuhong [2]. ECG has been known as the “ginseng of the South” since ancient times [3], and Hua Zhou Zhi (Qing Dynasty, A.D.1686) records that “a single genuine slice was worth one gold coin”. In 2024, the National Health Commission of China officially included ECG in the List of Substances with Both Medicinal and Edible Uses [4,5].

According to TCM theory, ECG resolves stagnation, dries dampness, resolves phlegm, regulates qi, and widens the middle burner. These traditional actions broadly parallel the modern pharmacological effects reported for ECG, such as its anti-inflammatory, antitussive, and expectorant activities. ECG contains several classes of constituents, including flavonoids, polysaccharides, volatile oils, and coumarins [6]. Flavonoids have received the most attention [7,8,9]. Naringin is the most abundant flavonoid and reportedly accounts for approximately 80% of the total flavonoid content [10,11].

ECG was officially designated a medicinal and edible substance in China [12]. Its nutritional value, bioavailability, and bioactivity have attracted increasing attention. However, most studies have focused on the effects of individual compounds, such as naringin and naringenin, and have rarely examined ECG as a whole [9,13]. This review therefore summarizes the botany, geographic distribution, phytochemistry, pharmacological activities, product applications, and patent information of ECG. It also provides an update on current knowledge of its chemical constituents and pharmacodynamic activities. It further discusses recent advances and outlines directions for future research, providing a scientific basis for the rational use of ECG.

## 2. Methodology

**Figure 1:**
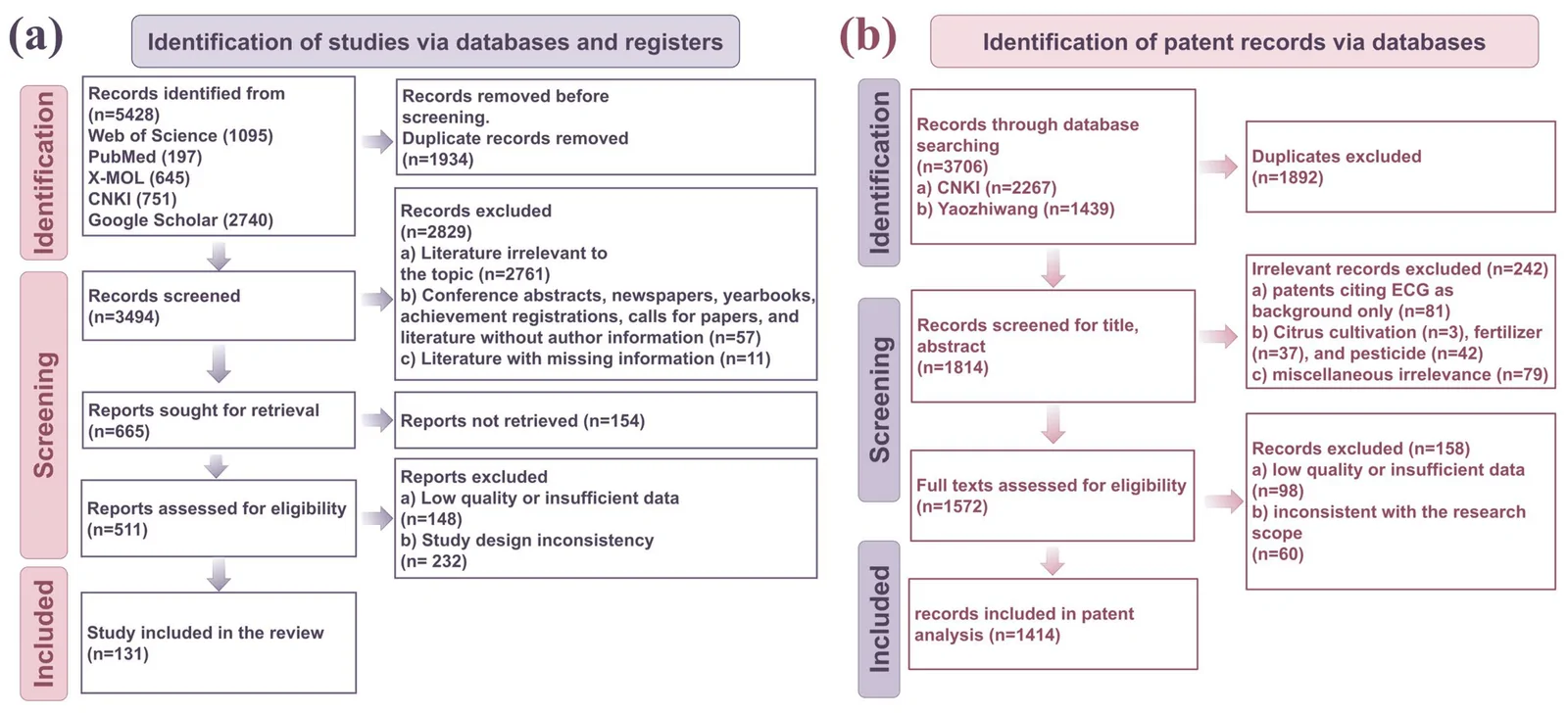
PRISMA flow diagram for literature and patent screening (flowchart of literature retrieval and screening (**a**); flowchart of patent retrieval and screening (**b**)).

This review covers published studies concerning ECG up to 2026. A comprehensive search of Web of Science, PubMed, X-MOL, Google Scholar, and CNKI was performed using the following search terms: “*Exocarpium Citri Grandis*”, “Huajuhong”, “*Citrus grandis* ‘Tomentosa’”, “*Citrus grandis* (L.) Osbeck”, “pomelo peel”, “phytochemistry”, “pharmacology”, “chemical constituents”, “biological activity”, “application” and “extraction”. The initial search returned 5428 records. Duplicates were first identified automatically on the basis of title, author and publication year and were then cross-checked manually. After removal of 1934 duplicates, 3494 unique records were left for abstract screening. At this stage, irrelevant, non-peer-reviewed, and incomplete records were excluded. Full texts of 665 potentially eligible reports were retrieved, of which 154 were unavailable. Subsequent eligibility assessment of the remaining 511 reports excluded low-quality studies and those with inconsistent designs, leaving 131 studies for this review. The literature screening workflow is presented in Figure 1a. English publications were given priority, while non-English studies were also considered when they provided reliable bibliographic information, English abstracts, or accessible full texts.

Furthermore, we consulted information from the Pharmacopoeia of the People’s Republic of China (Chinese Pharmacopoeia, 2025 Edition) and ancient classical books. The botanical characterization presented herein was sourced from the Flora of China database (https://www.iplant.cn/info, accessed on 5 July 2026). The traditional prescription information was obtained from Yaozhiwang (https://www.yaozh.com, accessed on 10 July 2026). Patent information was obtained from CNKI and Yaozhiwang (https://www.yaozh.com, accessed on 19 July 2026) using the following keywords: “*Exocarpium Citri Grandis*”, “Huajuhong”, “*Citrus grandis* ‘Tomentosa’”, “*Citrus grandis* (L.) Osbeck”, “pomelo peel”, and “Juhong Tai”. The inclusion and exclusion criteria for patents are detailed in Figure 1b. CAS SciFinder (https://scifinder-n.cas.org/, accessed on 8 August 2026), PubChem (https://pubchem.ncbi.nlm.nih.gov/, accessed on 8 August 2026) and ChemSpider (https://www.chemspider.com/, accessed on 8 August 2026) were used to verify the chemical structures of compounds reported in the literature.

## 3. Botany

### 3.1. Botanical Origin

**Figure 2:**
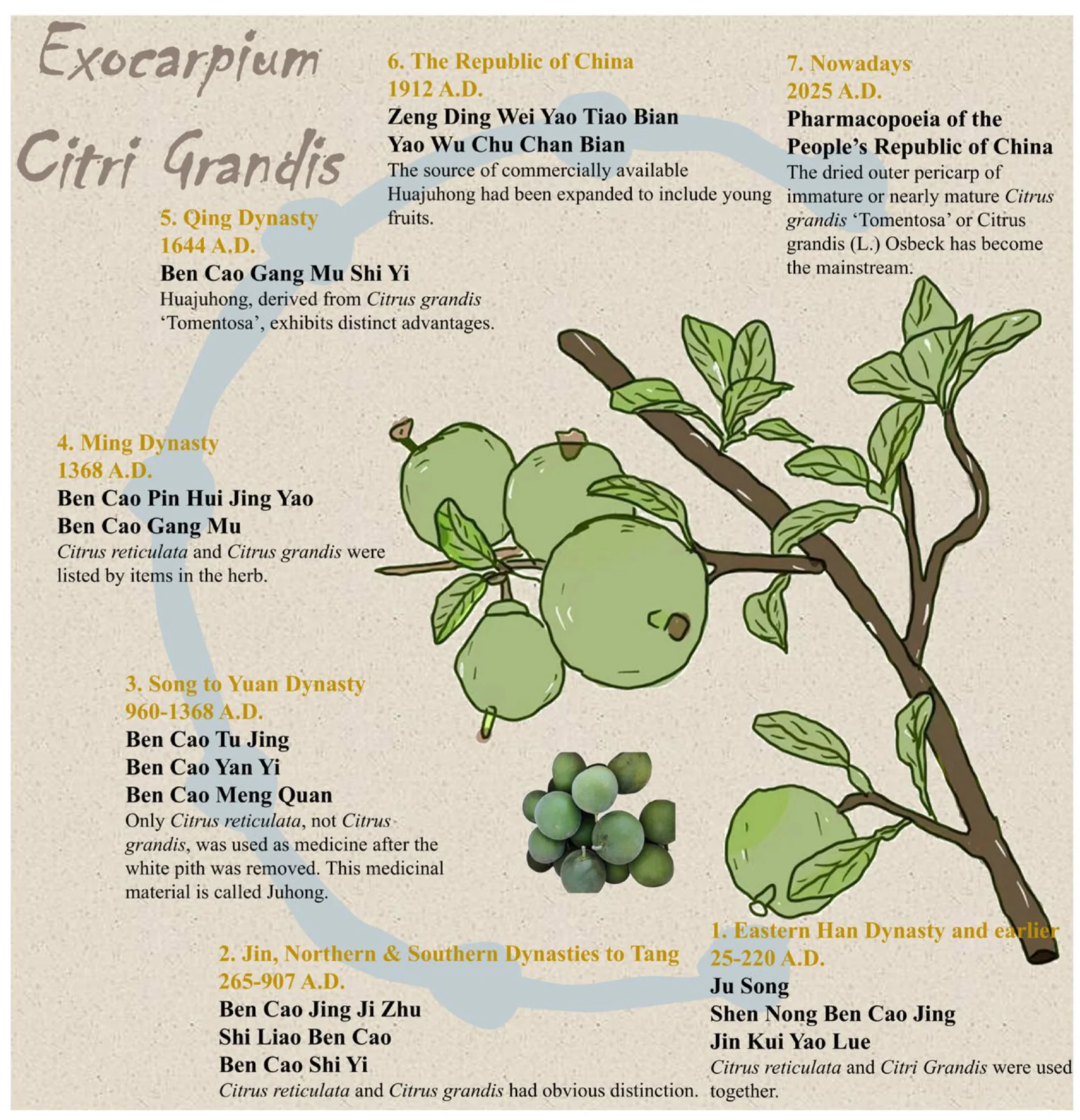
The medicinal history timeline of ECG.

There are variations in the nomenclature, botanical origin, and medicinal parts of ECG across historical periods [2]. This review compiles information on ECG from historical herbal works and pharmacopeias (Figure 2). Historically, Huajuhong encompassed the following two types: citrus-derived juhong and pomelo-derived juhong, which correspond to *Citri Exocarpium Rubrum* (Juhong) and *Exocarpium Citri Grandis* (Huajuhong) in the Chinese Pharmacopoeia (2025 Edition), respectively. The name “Huajuhong” first appeared in Shi Yao Wei Bian [14], and it also bears aliases including “hua pi”, “hua chen” [15]. Its processed decoction pieces are known by local names such as “maojuhong”, “seven-claw”, “six-claw”, and “five-claw”. The character “hua” in “Huajuhong” denotes its authentic origin in Huazhou City and served as an authenticity marker [16]. This regional designation functioned as a marker of genuineness in ancient medicinal trade. The earliest written record of ECG as a medicinal material was found in Guang Dong Tong zhi (1675), and Hua Zhou Zhi (1748) documented that ECG originated from *Citrus grandis* “Tomentosa”, rather than *Citrus reticulata* Blanco [17]. ECG was first recorded as an independent entry in Ben Cao Gang Mu Shi Yi in 1765 [18].

In modern times, the Chinese Pharmacopoeia (1963 Edition) placed Huajuhong under the Juhong monograph, while the 1977 edition retained only Juhong and removed Huajuhong [19]. Since the 1985 edition, Huajuhong has had its own monograph, separating from Juhong, in all subsequent editions [20]. Currently, the 2025 edition of the Chinese Pharmacopoeia defines ECG as the dried outer pericarp of immature or nearly mature fruits of *Citrus grandis* “Tomentosa” (CGT) or *Citrus grandis* (L.) Osbeck (CGO) [21] (Table 1). Although the Chinese Pharmacopoeia accepts both CGT and CGO as official botanical sources of ECG, the two taxa are not equivalent. CGT is a hairy-peeled cultivar of *Citrus maxima* and represents the traditional authentic source, whereas CGO serves merely as an alternative source. Because their medicinal quality differs, reliable analytical methods are needed to differentiate these two taxa for quality control.

### 3.2. Botanical Characterization and Distribution

There are differences in botanical characteristics, morphological traits, and geographical distribution between CGT and CGO. CGO is also known as *Citrus maxima* (Burm.) Merr. CGO is an arbor that predominantly grows in humid tropical biomes. The fruits are globular, oblate, pyriform, or broadly conical in shape. Its transverse diameter is usually greater than 10 cm. Fruit color is light yellow or yellow-green, while hybrid varieties may display a vermilion hue. Its pericarp is either thick or thin, spongy, with large, raised oil cells, and the fruit core is solid yet soft. CGO bears 10–15 locules, with a maximum of 19 locules. It may contain over 200 seeds, although some varieties are seedless. Flowering occurs between April and May, with fruiting taking place from September through December. In China, CGO is predominantly distributed south of the Yangtze River. Its northernmost distribution occurs in the Xinyang and Nanyang areas of Henan Province, all of which are cultivated. Globally, this species stretches from Assam to Indo-China. It is cultivated in southeast Asian countries such as Cambodia, Laos, Myanmar, Thailand, and Vietnam (https://powo.science.kew.org, accessed on 5 July 2026).

CGT is a cultivated variety unique to Huazhou, Guangdong Province [17]. Although CGO has long been accepted as an alternative source, the two botanical origins differ appreciably in quality and medicinal value [22]. CGT is nonetheless regarded as superior, largely owing to the region’s distinctive ecological and edaphic conditions [5]. The overall plant morphology and leaf shape of CGT are similar to those of other *Citrus maxima* plants in the same genus. But there are significant differences in fruit morphology. The fruit of CGT is a subglobose hesperidium. The mature fruit presents as lemon yellow, while the immature fruit is green. The pericarp is densely covered with thick pubescence. The pericarp has an extremely thick albedo layer that is not easily separable from the pulp. There are 16 locules. The pulp is sour and bitter, unfit for raw consumption; the fruit contains numerous seeds [23]. CGT predominantly occurs in the tropical and subtropical zones of southern China, including Guangdong, Guangxi, Yunnan and other areas [20]. Although the Chinese Pharmacopoeia (2025 Edition) recognizes CGT as a separate botanical source of ECG, CGT is not accepted as a distinct species in Flora of China or POWO, where it is treated as a cultivar within Citrus maxima. Because species-level DNA barcodes have limited resolution within Citrus, authentication of CGT must rely on combined evidence (pericarp pubescence, cultivar-level molecular fingerprints, and diagnostic chemical markers) rather than species identification alone.

## 4. Ethnopharmacology

### 4.1. Traditional Uses

ECG is pungent and bitter in taste and warm in nature. The pungent property promotes circulation and dispersion, while the bitter property purges fire and dries dampness [24]. Yao Pin Hua Yi (Ming Dynasty, 1624 AD) recorded that coughs with sputum should be treated by regulating qi. ECG excels at removing phlegm accumulated in the lungs and spleen and is frequently combined with Jiaosanxian for pediatric coughs to resolve food retention and dissipate phlegm [25]. Compared with CGO, CGT exhibits relatively stronger effects in resolving phlegm and relieving coughs. This distinction arises from the unique ecological environment of Guangdong Province, which endows CGT with warm and drying properties [22,26]. ECG can also aid digestion, alleviate greasiness, relieve alcohol intoxication, and neutralize crab toxicity. Additionally, Ben Cao Gang Mu recorded that ECG relieves stagnant qi, and its therapeutic potency increases with storage duration. Nevertheless, ECG should not be used indiscriminately, as its warm and drying nature may deplete body fluids and injure yin-fluid when used inappropriately or excessively [24].

### 4.2. TCM Prescriptions

A TCM prescription consists of a therapeutic combination of multiple herbs. According to their importance and functions, medicinal herbs within the prescription are divided into “Monarch, Minister, Assistant, and Envoy”. ECG is mainly used in formulas for resolving phlegm and suppressing cough, including Zhike Juhong Ye, Juhong Wan, Juhong Pian, Jubei Banxia Tang, and so on. These formulas are prepared in diverse dosage forms, including tablets, pills, capsules, granules, decoction, mixture, and syrup. This review summarizes 18 classic formulas containing ECG, which helps understand the traditional medication habits of ECG (Table 2).

### 4.3. Harvesting and Processing

The harvesting of ECG differs somewhat between the two source plants (CGT and CGO). For CGT, harvested fruits are divided into two categories. The first category comprises young fruits: fallen fruits collected from April to May are processed into maojuhong tai (young fruit of CGT). Uniform size and abundant pubescence are desirable traits. The second category consists of nearly mature fruits harvested in June–July, for which turquoise color and dense pubescence are preferred. According to the fruit morphology and the density of pubescence on the fruit surface, these materials are further classified into zheng-maojuhong (high-grade CGT with dense pubescence) and fu-maojuhong (lower-grade CGT with relatively sparse pubescence). For CGO, ECG is obtained either from fallen fruits harvested in May or from the outer pericarp of mature fruits in November [27].

**Figure 3:**
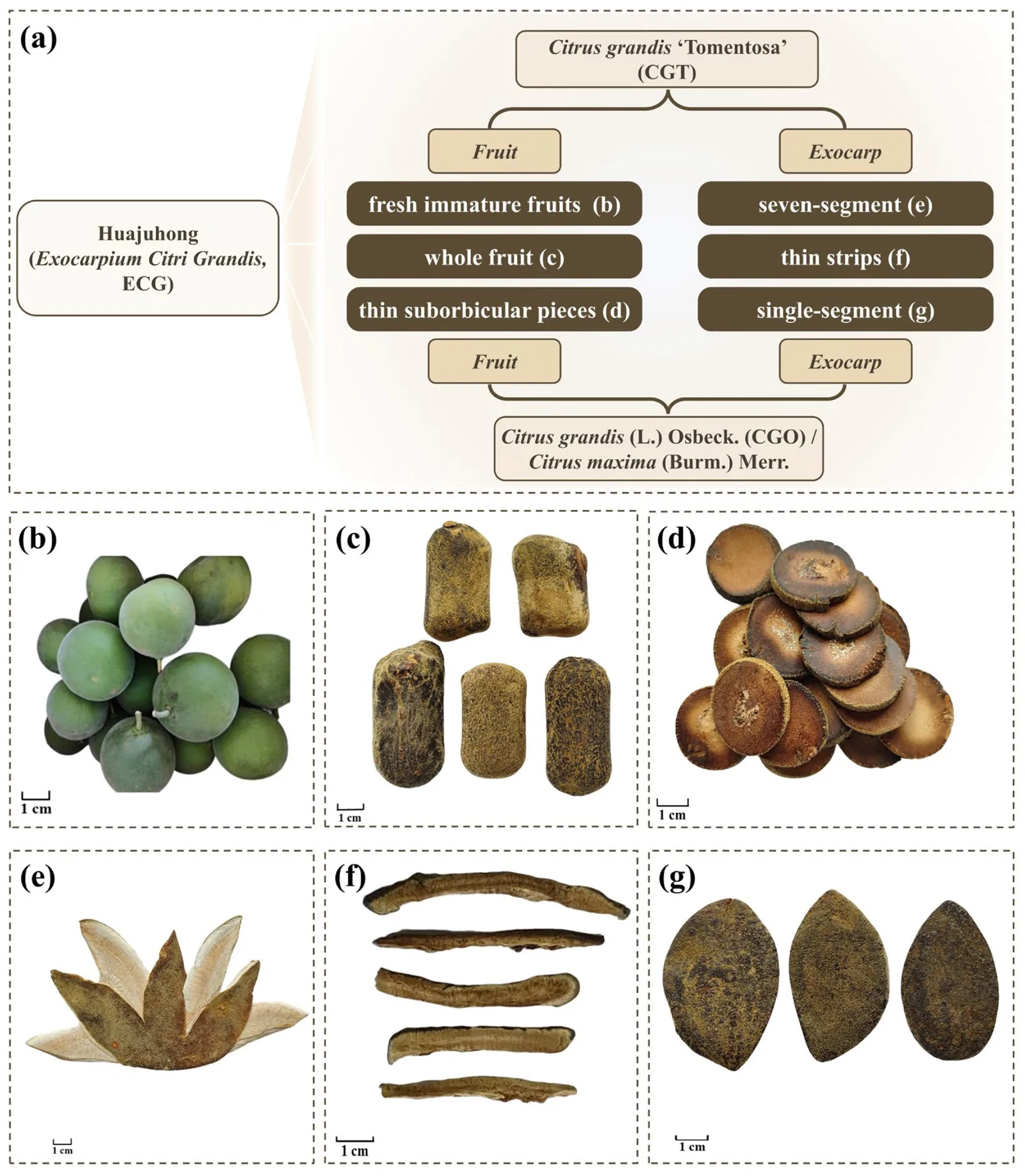
Different commercial specifications of ECG (illustration showing the relationship among various ECG commercial specifications (**a**); fresh immature fruits (**b**); whole fruit (**c**); thin suborbicular pieces (**d**); seven-segment (**e**); thin strips (**f**); single-segment (**g**)).

After harvesting, fresh fruits of CGT are blanched in hot water at 80 °C and subsequently dried either by sun exposure or baking [28]. This process is not required for CGO. Multiple commercial specifications of ECG exist on the market (Figure 3)**,** including intact outer pericarp, thin strips or suborbicular pieces [22], and a decoction-piece specification named maojuhong tai pian [22,29]. This product is prepared by pressing and slicing. Immature fruits possess a firm texture, which makes thorough moistening of the inner tissue difficult [18]. Accordingly, novel softening and drying procedures for ECG decoction pieces have been developed. The following three softening approaches were tested: decompression water moistening, steam moistening and airtight moistening. The steam moistening method was the most effective. The following three drying techniques were evaluated: constant-temperature drying, vacuum drying and microwave drying. The content of marker components in ECG increased after microwave drying [30], suggesting that softening and drying strategies exert a notable influence on the quality control of ECG.

## 5. Phytonutrients

### 5.1. Amino Acids

Although amino acids are present in plants at relatively low concentrations, they are indispensable for maintaining physiological nitrogen balance [31]. Table 3 summarizes the profiles and concentrations of amino acids in fruits, flowers and leaves of ECG. For essential amino acids, ECG flowers contain higher levels of these, including L-threonine, phenylalanine, valine, L-lysine, DL-tryptophan and L-isoleucine, compared with fruits and leaves, while L-histidine is most abundant in leaves. L-methionine is not detected (ND) in flowers, indicating the absence of this sulfur-containing essential amino acid within this tissue. Among non-essential amino acids, proline exhibits strong tissue specificity. Leaves contain proline at 59.48 mg/100 mL, which is higher than that in flowers (19.58 mg/100 mL) and fruits (5.69 mg/100 mL). As proline is a well-known stress-responsive metabolite in plants [32], its high accumulation in ECG leaves suggests an adaptive response to environmental stress, and leaves can serve as a rich natural source of proline. In contrast, aspartic acid, L-alanine, DL-serine and arginine reached their maximum concentrations in flowers, demonstrating that flowers are the primary tissue accumulating most free amino acids (except proline). 4-aminobutanoic acid (GABA), a bioactive non-protein amino acid [33], occurs at higher levels in leaves than in fruits, suggesting that leaves have potential for use in functional amino acid products. Overall, amino acid profiles differ substantially across ECG tissues. Flowers accumulate abundant essential amino acids and most non-essential amino acids; leaves are characterized by extremely high proline content; and fruits show relatively low total amino acid levels. This distinct spatial distribution of amino acids provides a chemical basis for bioactivity assessment, nutritional evaluation, and differentiated utilization of different ECG tissues [34].

### 5.2. Minerals

Mineral elements are essential components for plant physiological processes and also contribute to the nutritional value of herbal materials [35]. Table 4 summarizes the mineral profiles in the peels, pulps and whole fruits of ECG. Among the detected mineral elements, K was the most abundant element in both peels and pulps, while the whole fruits exhibited the highest cumulative content of the determined mineral elements (376.00 μg/kg), followed by peels (326.25 μg/kg) and pulps (317.36 μg/kg). K accumulates preferentially in pulps, whereas Cu, P, Mg, Fe, Zn and Na are more abundant in peels. In contrast, Mn and Ca show higher levels in the whole fruits compared with isolated peel and pulp fractions. These distinct distribution patterns of mineral elements in ECG tissues reflect tissue-specific accumulation of inorganic nutrients. These findings provide chemical evidence supporting the nutritional assessment and differentiated utilization of ECG peels, pulps, and whole fruits [36].

## 6. Phytochemistry

### 6.1. Preparation

**Figure 4:**
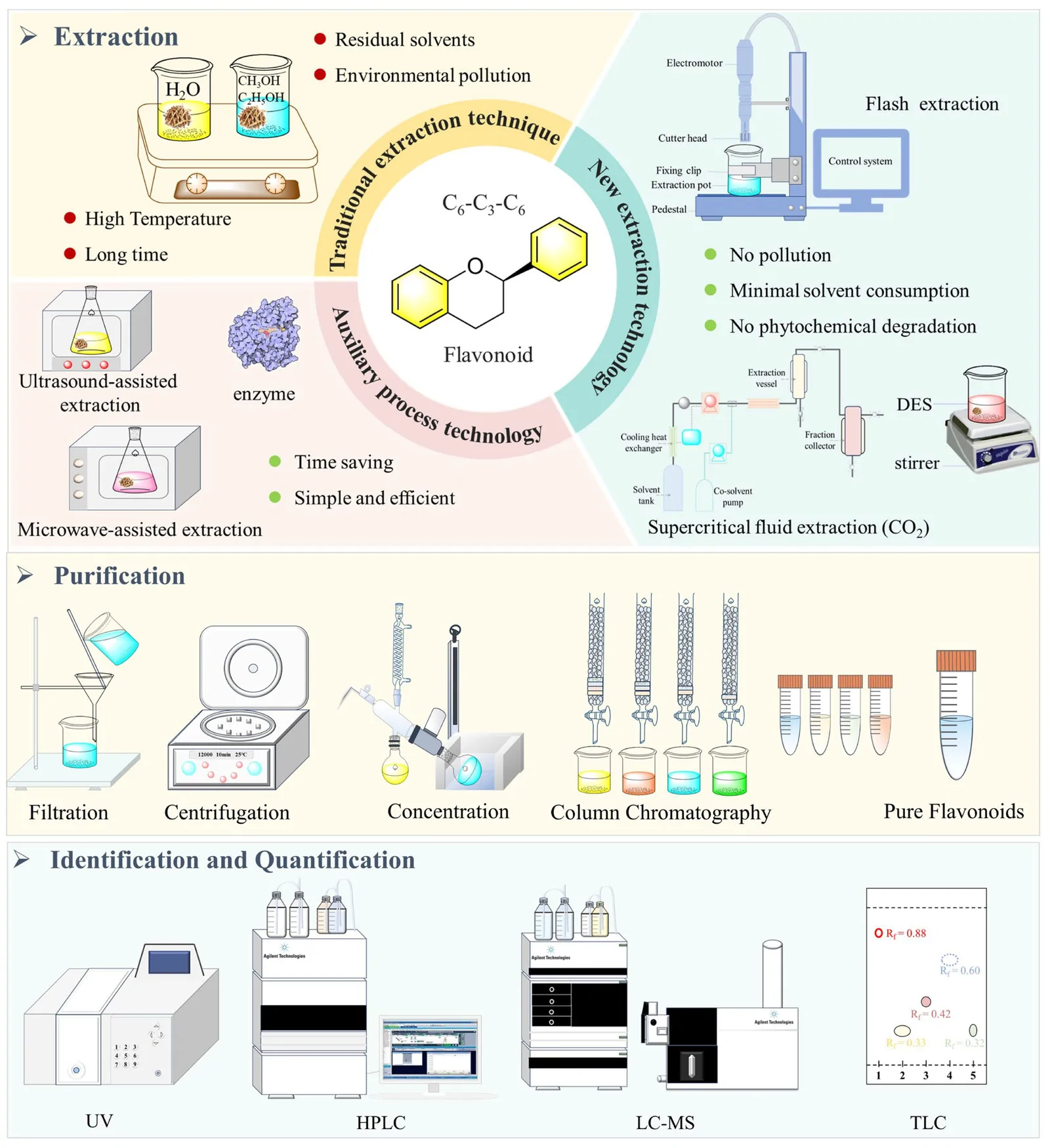
Extraction, purification and separation procedures of flavonoid components in ECG (the features indicated by green and red circles represent advantages and disadvantages respectively).

Adding plant-derived active ingredients to food products is a growing trend [38,39,40]. ECG has a long history of medicinal use and broad application value [41]. Its therapeutic effects are mainly attributed to the flavonoids [42]. Therefore, this review describes the separation, purification, characterization, and quantification of flavonoid constituents (Figure 4).

#### 6.1.1. Extraction Methods

Extraction is the primary step in recovering bioactive components from plants [43,44,45]. Appropriate extraction techniques are critical to maximize yield. The following three categories of methods are used for flavonoid extraction from ECG: conventional, auxiliary-assisted, and novel methods. This subsection summarizes these strategies, including their applications, strengths and limitations. The main extraction methods for ECG flavonoids are listed in Table 5.

##### Conventional Extraction

Conventional extraction (CE) of ECG flavonoids mainly relies on solvent leaching and reflux [59]. Ethanol immersion achieved a flavonoid extraction rate of 3.19% [47]. Soxhlet extraction was used to obtain naringin from ECG samples of different ages, with an average naringin content of 11.98% [46]. The naringin content in aqueous ECG extracts reached 56.15 mg/g [48]. Ethanol-based extraction is versatile and widely adopted for ECG flavonoids extraction. However, this approach generally requires elevated temperature and prolonged extraction time to obtain satisfactory yields, which leads to several drawbacks as follows: long processing times, complicated procedures, and potential degradation of thermolabile constituents [60,61].

##### Auxiliary Process Technology

Microwave-, ultrasonic-, and enzyme-assisted technologies are widely combined with CE to improve performance [62]. Microwave-assisted extraction directly excites solvent molecules to generate rapid vibration and heat. A comparative study of Soxhlet, microwave and ultrasonic extraction confirmed that microwave-assisted extraction achieved the highest recoveries, with 80.56% polyphenols and 86.58% flavonoids [63]. Ultrasound has also been coupled with novel solvents for ECG extraction [64]. The ultrasonic-assisted natural deep eutectic solvent extraction of flavonoids from ECG was optimized, with the maximum total flavonoids (TF) content reaching 17.999 mg/g under conditions of 15 min and 60 °C [57]. Additionally, the complex enzyme-assisted ultrasonic method was used to extract TF from ECG, with an extraction rate of 1.80% [54]. Notably, the TF extraction rate varies with ethanol concentration. The highest rate occurs at an ethanol concentration of 95% [65]. Enzymatic treatment is another effective auxiliary approach [66]. Under optimized conditions (0.32% enzyme dosage, pH 4.83, 30 min shaking hydrolysis), compound enzyme-assisted extraction yielded TF of 51.03 mg/mL from ECG. [67]. The ratio of composite enzymes also modulated extraction efficiency, and adjusting the ratio from 1:2 to 2:1 increased the yield to 1.80% [54].

##### Other Novel Extraction Methods

In recent years, supercritical fluid extraction (SFE) technology has served as a promising alternative to CE [68]. Supercritical carbon dioxide (SC-CO_2_) extraction of flavonoids from ECG afforded a yield of 2.37%, and the obtained flavonoids exhibited stronger free radical scavenging activity than extracts obtained by CE [69]. Deep eutectic solvents (DESs) consist of ionic liquids and low-molecular-weight compounds, and they can form hydrogen or ionic bonds with flavonoids to enable efficient extraction [70]. Response surface methodology (RSM) has been used to optimize DES extraction of ECG flavonoids, and the choline chloride–ethylene glycol system achieved the highest flavonoids yield of 19.31 mg/g [56]. Compared with CE, DES extraction offers simple operation, environmental compatibility, high solubilization capacity and convenient solvent recovery [71].

In summary, these performance differences among extraction approaches are closely related to solvent properties and the physicochemical characteristics of compounds. CE suffers from low efficiency and poor sustainability. Auxiliary technologies improve yield and selectivity while shortening extraction duration and mitigating thermal degradation. Novel extraction strategies provide eco-friendly alternatives and preserve flavonoid bioactivity and thus hold great application potential.

#### 6.1.2. Purification Methods

ECG extracts contain complex matrices, so further refinement is required to obtain pure flavonoids [11]. Purification of ECG flavonoids can be divided into crude purification and fine purification. Ordinarily, crude purification is achieved by filtration and centrifugation [72]. Fine purification aims to fractionate crude extracts for subsequent isolation. Representative fine purification techniques for ECG flavonoids include macroporous resin adsorption (MRD), molecular imprinting technology (MIT), and high-performance counter-current chromatography (HPCCC). MRD is widely applied for the separation of natural products [73]. AB-8 macroporous resin was used to purify ECG ethanol extracts. Sequential elution with distilled water and 50% ethanol was performed; the 50% ethanol eluate was collected, concentrated and vacuum-dried prior to spectrophotometric determination of TF [74]. MRD features simple operation, low cost and easy industrial scale-up. Nevertheless, its selectivity is affected by resin type, operating parameters and structural differences among flavonoids [75,76]. A comparison of five resins (LR-20, D-312, DM-130, CAD-40 and AB-8) for ECG flavonoids purification demonstrated that D-312 gave the optimal performance [77]. MIT is a highly selective purification technology. A molecularly imprinted polymer for naringin was fabricated to isolate naringin from ECG, showing high selectivity, good stability and reusability [78]. HPCCC is a liquid–liquid partition-based chromatographic method with high separation efficiency and is suitable for separating complex mixtures. HPCCC processed 50 mg crude extract to yield 28.8 mg naringin (97.1% purity) and 1.4 mg rhoifolin (95.5% purity) [79]. It has also been adopted to separate naringin and its aglycone naringenin from ECG; 83.3 mg naringin (>99% purity) and 27.5 mg naringenin were recovered from 1 g flavonoids extract [80].

Among these fine purification techniques, MRD is suitable for bulk enrichment of total flavonoids at low cost but provides limited resolution for individual flavonoid analogs. MIT achieves superior target-specific selectivity yet is limited to predefined template molecules. HPCCC can separate multiple flavonoids simultaneously and delivers high product purity, although it suffers from lower sample loading capacity and higher operational cost. In summary, multiple strategies are available for purifying ECG flavonoids, each with distinct merits and applicable scenarios. In practice, method selection depends on flavonoid properties, purification targets and production costs.

#### 6.1.3. Identification and Quantification Methods

It is essential to clarify the structures and classification of flavonoids in ECG to support further quality control. Multiple analytical approaches are applied to the identification and quantification of flavonoids in ECG (Table 6). Common techniques include high-performance liquid chromatography (HPLC), reversed-phase high-performance liquid chromatography (RP-HPLC), thin-layer chromatography (TLC), liquid chromatography–mass spectrometry (LC/MS), and nuclear magnetic resonance (NMR).

Electrospray ionization mass spectrometry (ESI-MS) is widely used for natural product analysis [83]. ESI-MS identifies 22 compounds in ECG, among which two C-glucosyl flavonoids were tentatively identified for the first time [84]. An analytical strategy based on ultra-fast liquid chromatography coupled with diode array detection and quadrupole/time-of-flight mass spectrometry (UFLC-DAD-Q-TOF-MS/MS) has been established for comprehensive and rapid quality evaluation of ECG, revealing that flavonoids and coumarins were abundant in both epicarp and whole fruit samples, and whole fruit possessed higher flavonoid levels than epicarp [85]. Thin-layer chromatography (TLC) enables the separation and visualization of characteristic flavonoid spots. TLC was used to determine the rhoifolin content in ECG and to compare it between maojuhong and guangjuhong [86]. Near-infrared spectroscopy (NIR) allows the simultaneous determination of multiple components, and it enables rapid high-throughput measurement of naringin in ECG [87,88,89]. Ultraviolet spectrophotometry (UV) is widely adopted for the quantitative analysis of ECG components. Single-wavelength and dual-wavelength UV spectrophotometers were used to determine the content of TF in ECG, with the outcomes indicating that the content ranged from 4.39% to 15.92% [90,91,92].

### 6.2. Structure and Classification

**Figure 5:**
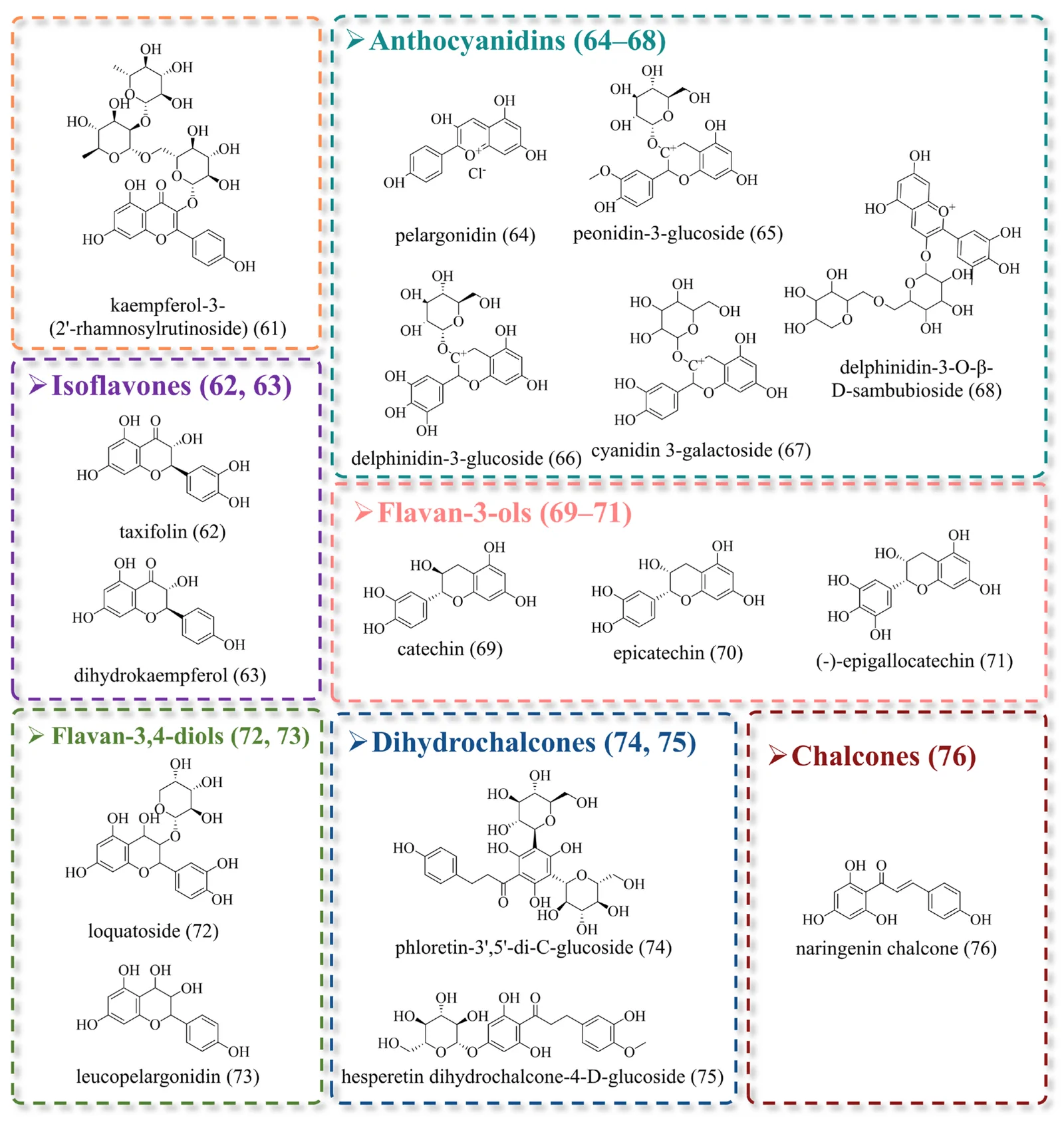

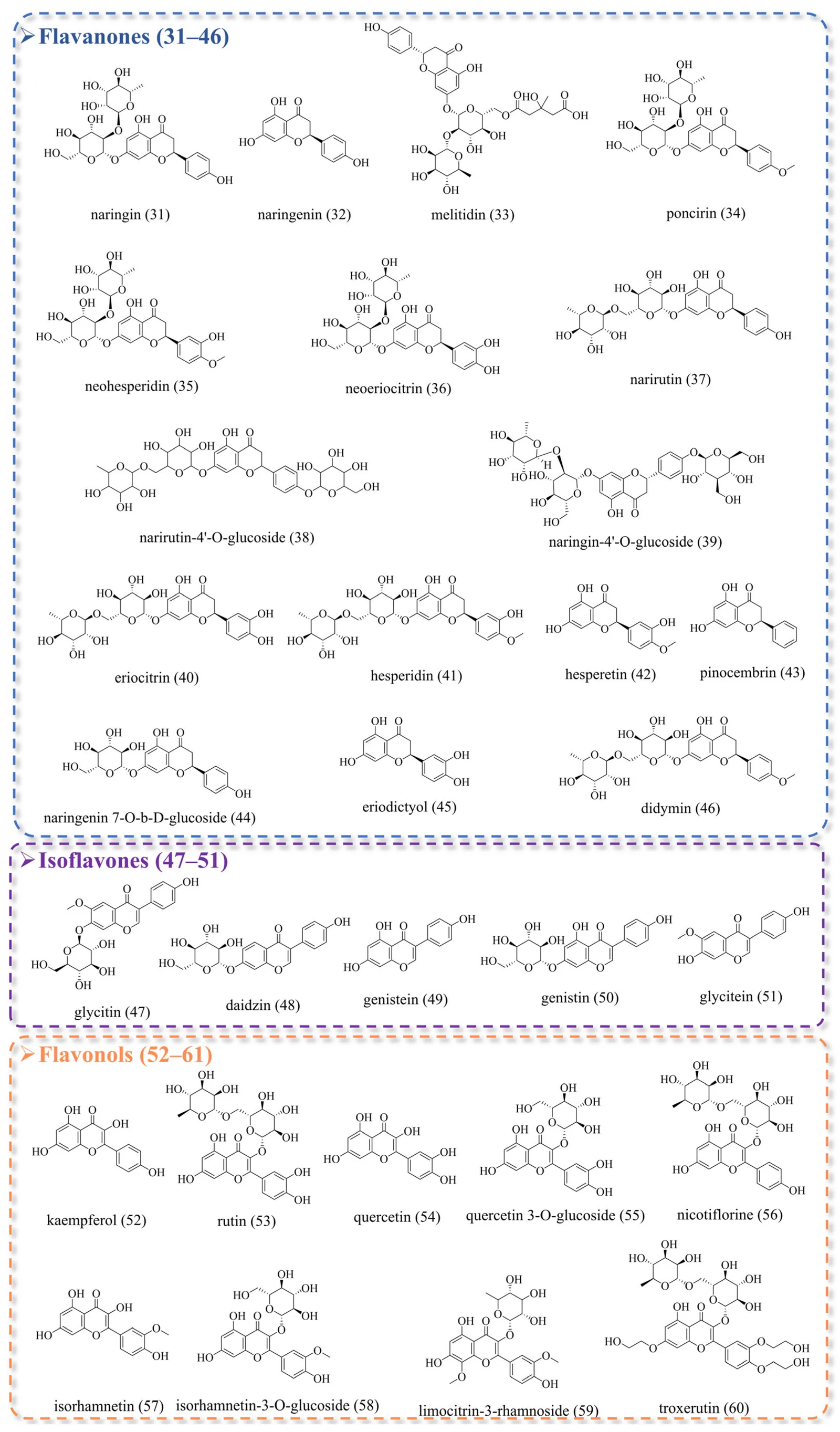

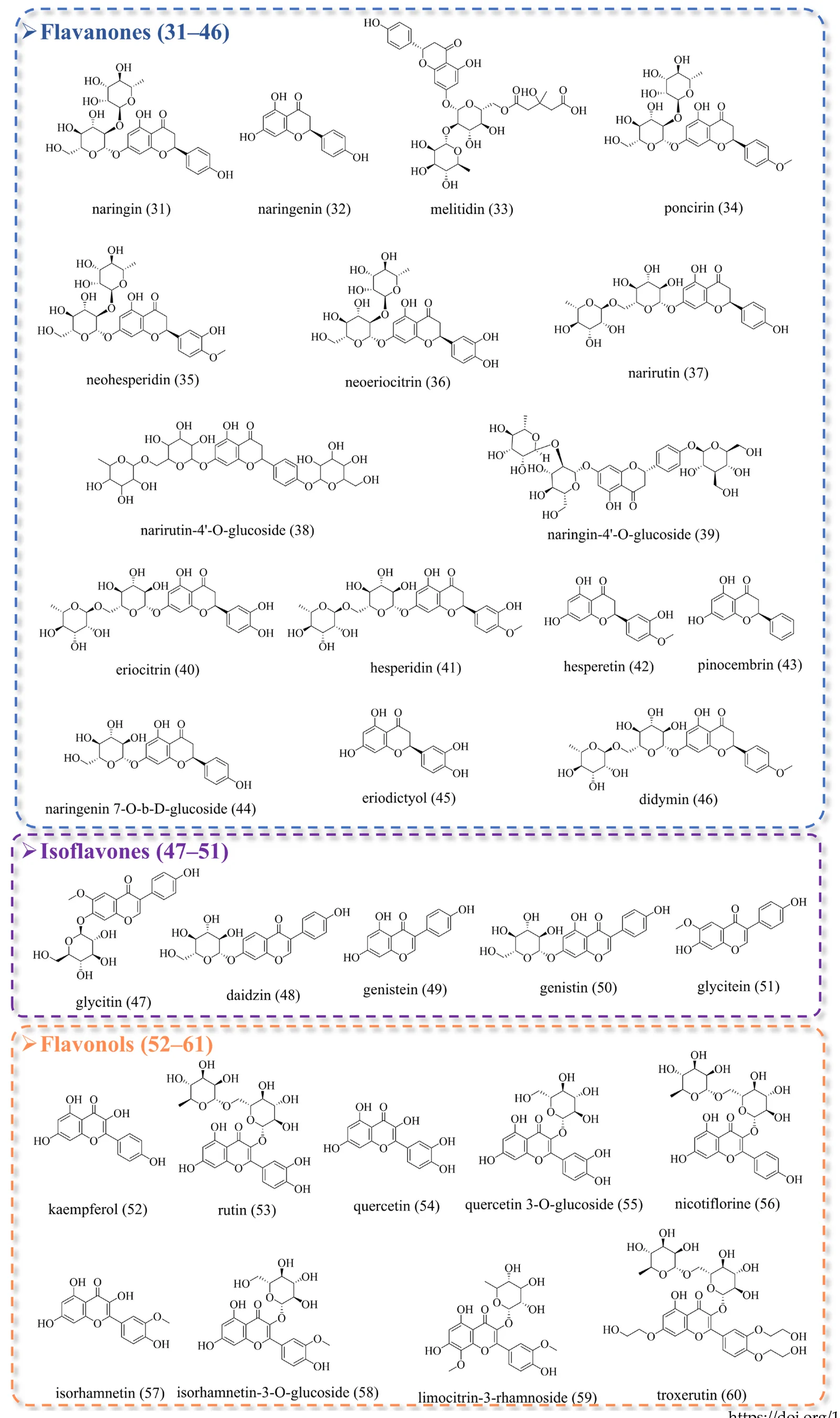
The chemical structures of flavonoids reported from ECG.

Flavonoids are categorized based on the oxidation level of the central three-carbon chain, the connection site of the B ring, and whether the three-carbon chain forms a ring or not [93]. The flavonoids contained in ECG mainly consist of flavones and flavanones [94]. This article summarizes 76 flavonoids annotated in ECG. They include 30 flavones (1–30), 16 flavanones (31–46), five isoflavones (47–51), 10 flavonols (52–61), two flavanonols (62, 63), five anthocyanidins (64–68), three flavan-3-ols (69–71), two flavan-3,4-diols (72, 73), two dihydrochalcones (74, 75), and one chalcone (76) (Figure 5). Among these, 13 compounds are unambiguously confirmed by authentic standards or NMR (Level 1), and 63 candidates are tentatively characterized by MS/MS and literature-based comparison (Level 2) (Table 7). Notably, most of these compounds lack definitive NMR structural confirmation. Further NMR structural confirmation represents an interesting direction for future research.

## 7. Biological Functions and Action Mechanisms

**Figure 6:**
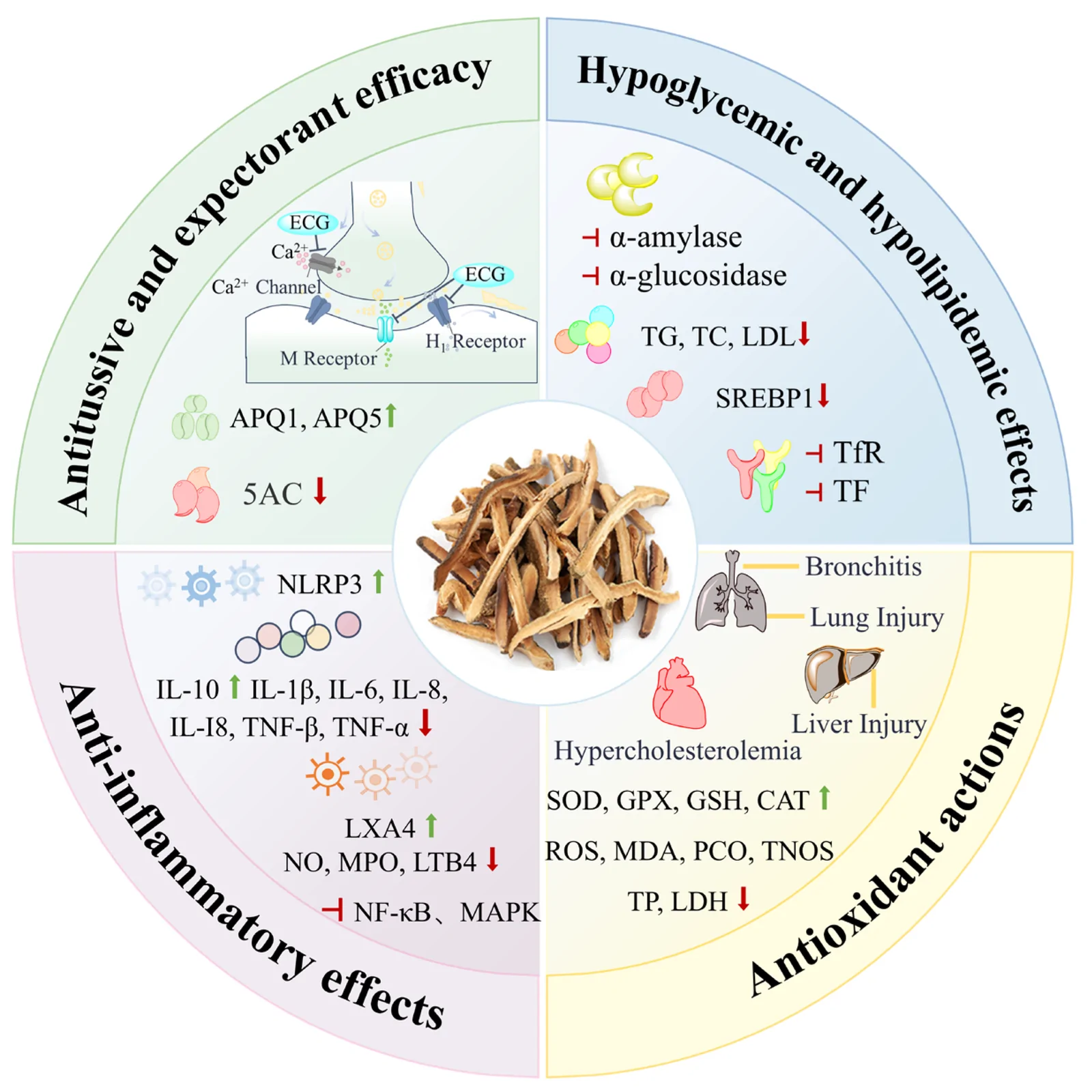
Biological functions and action mechanisms of ECG. (↑, increased or upregulated; ↓, decreased or downregulated; ⊣, inhibition.)

ECG is a widely used Chinese medicinal herb, and most of its traditional therapeutic effects have been elucidated by contemporary pharmacological research. It exerts antitussive, expectorant, anti-inflammatory, antioxidant, hypoglycemic, and hypolipidemic effects (Figure 6).

### 7.1. Antitussive and Expectorant Effects

Since the outbreak of the COVID-19 pandemic, TCM has played a notable role in managing respiratory symptoms associated with the infection [101]. ECG has garnered attention owing to its efficacy in relieving cough and reducing phlegm [102]. These effects are mediated by multiple mechanisms. In an in vitro assay using rat tracheal rings, ECG relieved smooth-muscle spasm by blocking calcium channels and antagonizing M and H_1_ receptors [103]. In the same model, ECG also inhibited bronchial smooth-muscle proliferation and stimulated the secretory activity of the gastric and laryngeal mucosa as well as the respiratory glands, thus diluting the mucus in the respiratory tract. ECG enhanced the motility of the tracheal mucociliary system, degraded the viscous components in sputum, and reduced sputum viscosity to promote expectoration [7]. In LPS-treated rat models, ECG inhibited the synthesis of airway mucin 5AC, suppressed the abnormal proliferation of goblet cells, and reduced the excessive secretion of mucin. ECG also promoted the secretion of airway serosa and upregulated the expression of AQP1 and AQP5, facilitating the expectoration of sputum [7]. In addition, the main component of ECG is naringin, which has been incorporated into the recommended prescriptions of the “Diagnosis and Treatment Plan for Novel Coronavirus Infection”. Clinical observations suggested that naringin-containing formulations may relieve cough and improve sputum expectoration in patients with COVID-19 [7]. Collectively, ECG extracts and isolated components (primarily naringin) have demonstrated antitussive and expectorant effects in vitro and in animal models. However, current evidence is derived predominantly from preclinical studies, and well-designed clinical trials remain necessary to verify these effects in humans.

### 7.2. Hypoglycemic and Hypolipidemic Effects

Diabetes mellitus is a global chronic metabolic disease characterized by sustained hyperglycemia [104]. It has emerged as a global public health concern [105]. Dietary carbohydrate digestion occurs primarily in the small intestine, and inhibiting the activities of *α*-glucosidase and *α*-amylase can effectively reduce postprandial blood glucose levels [106]. Total flavonoids of ECG (TFECG) inhibited both *α*-glucosidase and *α*-amylase, thereby reducing blood glucose levels and stimulating insulin secretion. Additionally, TFECG alleviated dyslipidemia and decreased liver and kidney indices in diabetic mice [74].

Beyond dysregulated glucose metabolism, impaired lipid metabolism is another hallmark of diabetes. Simultaneous control of both hyperglycemia and hyperlipidemia is considered a promising strategy for diabetes management [104]. In the high-fat diet (HFD)-induced rat model, ECG extracts reduced the levels of triglycerides (TGs), total cholesterol (TC), and low-density lipoprotein cholesterol (LDL-C). This hypolipidemic effect was mediated through the PXR-CYP3A4/FXR-LXR*α* signaling pathway [107]. Furthermore, ECG attenuated the progression of non-alcoholic fatty liver disease (NAFLD) by reducing lipid accumulation and ameliorating iron metabolism disorders in mice. Mechanistically, ECG suppressed the expression of sterol regulatory element-binding protein 1 (SREBP1), thus alleviating hepatic lipid accumulation [108]. In addition, ECG inhibited the HFD-induced upregulation of six-transmembrane epithelial antigen of the prostate 3 (STEAP3). ECG also downregulated hepcidin expression and upregulated ferroportin 1 (Fpn1) expression in hepatocytes. These effects collectively contributed to improvements in lipid metabolism [108].

### 7.3. Anti-Inflammatory Effects

ECG extracts, fractions, and representative constituents may alleviate inflammatory reactions and associated symptoms [109]. In the RAW264.7 macrophage model, ECG extracts regulated the release of inflammatory factors through Toll-like receptors (TLRs) and the MAPK signaling pathway [4]. In animal studies, ECG reduced macrophage recruitment in the liver, thereby alleviating the inflammatory response [108]. As revealed by systems pharmacology approaches, ECG might contribute to the treatment of chronic bronchitis by suppressing TLR4- and MyD88-mediated inflammatory responses as well as Bcl-2-, Bax-, and Caspase-3-mediated apoptosis [110]. Moreover, TFECG exhibited anti-inflammatory effects. TFECG reduced leukocyte infiltration by lowering the counts of white blood cells (WBCs), lymphocytes (LYMPHs), neutrophils (NEUTs) and monocytes (MONOs). TFECG blocked the production of TNF-*α*, IL-1*β*, IL-6, and IL-18 after PM_2.5_ inhalation, thus alleviating acute lung inflammation in mice [42]. The same study also revealed that TFECG may modulate the release of inflammatory factors by regulating the NLRP3 inflammasome. In addition, naringin, the major flavonoid component of ECG, inhibited airway inflammation, particularly LPS-induced acute pulmonary inflammation. The mechanisms primarily involved regulating inflammatory factors and promoting inflammation resolution. Naringin reduced the levels of pro-inflammatory factors such as IL-8, LTB4, and TNF-*α*, as well as MPO activity. It attenuated the decline in levels of the anti-inflammatory factor IL-10 and alleviated neutrophil infiltration. Additionally, in a guinea pig model of chronic bronchitis induced by cigarette smoke, pretreatment with naringin prevented the reduction in LXA4 levels and further promoted airway inflammation resolution. Naringin mitigated pulmonary inflammation and lung tissue damage by regulating NO release and the metabolism of the pro-oxidant homocysteine [7,111].

### 7.4. Antioxidant Actions

Existing studies indicate that ECG extracts, fractions, and representative constituents may modulate oxidative stress at both chemical and biological levels. At the chemical level, ECG has been reported to prevent the oxidative deterioration of peanut oil, lard, and other edible oils [112]. TFECG exhibited antioxidant capacity in vitro, and this capacity correlated with purity. The crude polysaccharides from ECG exhibited antioxidant properties in chemical radical-scavenging assays, and this activity was influenced by the purification method employed [15]. The reducing capacity of each polysaccharide fraction followed the order: crude polysaccharides > alkaline polysaccharides > acidic polysaccharides > neutral polysaccharides. The relatively higher activity of the crude extract is typically attributed to the synergistic effects of co-extracted antioxidant constituents, such as polyphenols and flavonoids. In contrast, targeted purification techniques, such as DEAE-52 cellulose anion-exchange chromatography, can selectively isolate acidic polysaccharide fractions with enhanced DPPH and hydroxyl radical scavenging abilities, which may contribute to cellular protection against oxidative damage [15]. It should be noted that the above findings were obtained under chemical conditions and do not necessarily predict in vivo efficacy.

At the biological level, TFECG alleviated oxidative stress in PM_2.5_-induced acute lung injury models. Specifically, TFECG reduced the activities of lactate dehydrogenase (LDH) and inducible nitric oxide synthase (iNOS). TFECG also reduced total protein (TP) levels and promoted the activity of glutathione peroxidase (GSH-Px). It also increased the ratio of reduced glutathione (GSH) to oxidized glutathione (GSSG) [42]. Moreover, the major flavonoids in ECG exhibited antioxidant activity. In a guinea pig model of chronic bronchitis, naringin increased superoxide dismutase (SOD) activity in pulmonary tissue, suggesting that it may slow disease progression. In the HFD model, naringenin protected rats against hypercholesterolemia-induced cardiac oxidative stress. In this model, HFD elevated cardiac reactive oxygen species (ROS), malondialdehyde (MDA), lipid hydroperoxides (LOOHs), nitrite (NO_2_^−^), and protein carbonyls (PCO). HFD also reduced cardiac Na^+^, K^+^-ATPase activity and the activities of enzymatic antioxidants, including SOD, catalase (CAT), and glutathione peroxidase (GPx). In addition, HFD lowered the levels of non-enzymatic antioxidants, namely non-protein sulfhydryl (NPSH), GSH-Px, vitamin C, and vitamin E. Treatment with naringenin decreased these elevated cardiac oxidative markers (ROS, MDA, LOOH, NO_2_^−^ and PCO). Naringenin also partially restored cardiac antioxidant status [113]. It is important to note that all in vivo evidence cited above is derived from rodent or guinea pig models, and whether similar effects occur in humans has not been established.

Collectively, ECG and its bioactive compounds display diverse preclinical pharmacological activities (Table 8). These activities include antitussive and expectorant, glucose- and lipid-regulating, anti-inflammatory, and antioxidant effects, which involve modulation of smooth-muscle function, digestive enzymes, lipid-metabolic signaling, iron homeostasis, inflammatory-mediator release and endogenous antioxidant defense. However, the currently available data on ADME profiles and oral bioavailability of ECG and its principal constituents remain limited, most of which have been confined to short-term administration in rodents. These gaps constrain the extrapolation from animal models to humans.

## 8. Applications

### 8.1. The Applications of ECG in Industry, Food, and Medicine

**Figure 7:**
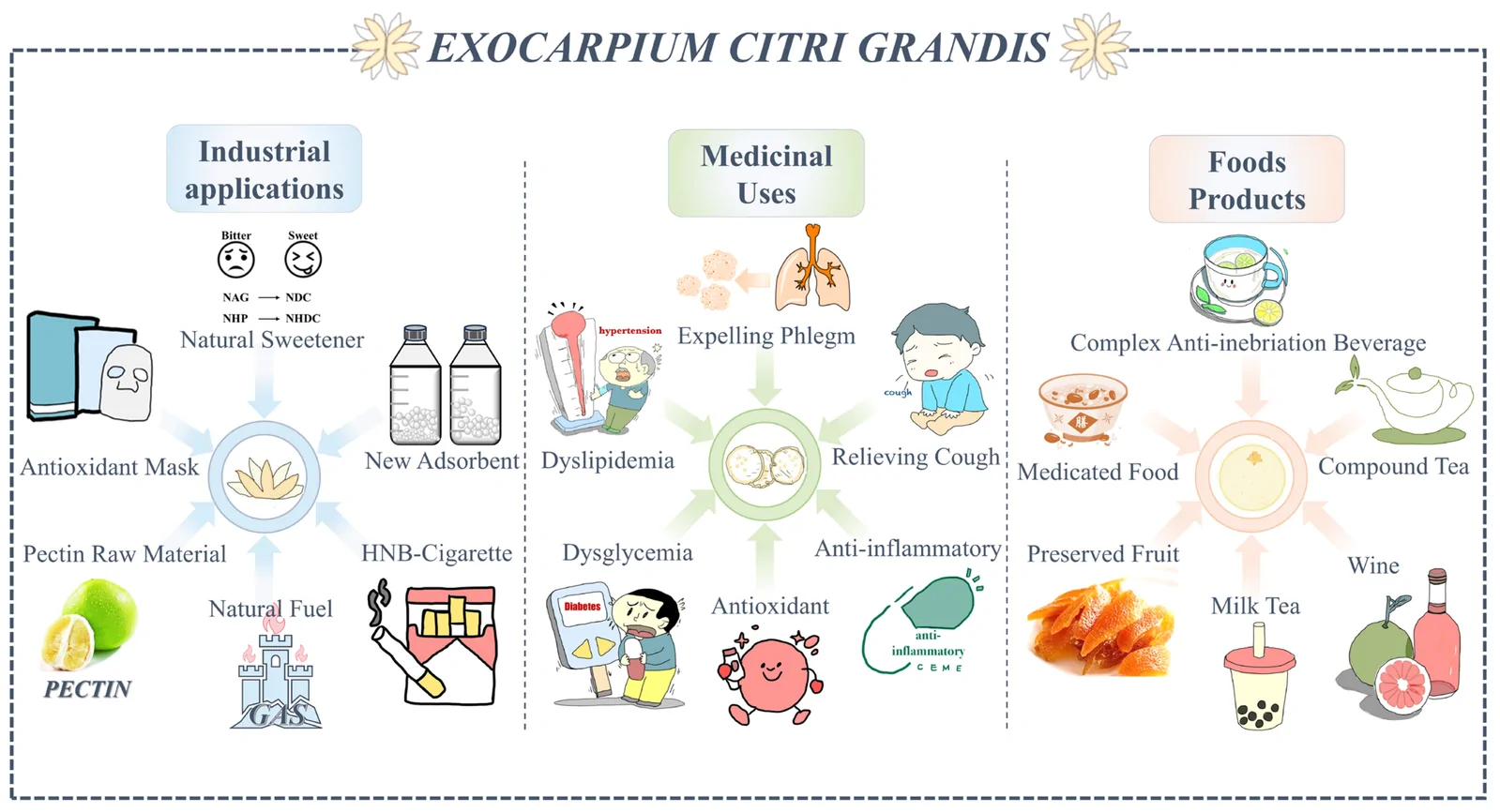
The main application of ECG, including industrial, medicinal, and food.

Medicinal and edible substances are herbal resources that serve as both food and medicine. As a geo-authentic herb from Huazhou, ECG has a cultivated area exceeding 0.87 ×10^4^ hm^2^ and over 200 processed products such as pharmaceuticals, decoction pieces, foods and beverages [119]. ECG obtained national geographical indication protection in 2006; its processing technique received provincial intangible cultural heritage in 2012. More importantly, ECG was included in the medicinal-edible substance catalog in 2024, enabling its dual-track development for medicinal and food applications (Figure 7). Rational exploitation of ECG can greatly increase the added value of its products [120]. Broadly, ECG applications fall into the following two distinct categories: commercially mature products and laboratory-stage exploratory studies.

In the industrial sector, ECG applications span a spectrum from well-established commercial products to early-stage laboratory investigations. Naringin and neohesperidin, the major flavonoids in ECG, can be hydrogenated into natural sweeteners. Notably, neohesperidin dihydrochalcone (NHDC) is 1000 times as sweet as sucrose. It has been approved under the EU Sweeteners Directive since 1994, finding wide use in beverages, dairy products, and confectionery [121,122]. NHDC thus represents a commercially mature product derived from ECG. In contrast, immobilized *α*-L-rhamnosidase has been employed as a catalyst with naringin as the raw material to enzymatically produce another natural sweetener (trilobatin) [81]. In addition, ECG can act as an adsorbent for removing heavy metals and RB114 dye from aqueous solutions [123] and serve as a feedstock for producing biofuels such as ethanol and methane [124]. Nevertheless, both adsorption and biofuel conversion applications of ECG are still confined to laboratory investigations and have not been scaled up for industrial use.

ECG has extensive applications in the food sector, most of which have been commercialized, while a few novel formulations remain at the development stage. Fresh ECG fruits have a sweet and sour taste with a refreshing fruity aroma and their flesh can be eaten directly. Dried fruits are used for making tea, which has a sour and slightly bitter taste, and are best consumed after meals or with alcohol. ECG also serves as an ingredient in manufacturing various products, including milk tea and candied fruits [125]. Fengquan Winery in Huazhou was the first to use ECG to make Juhong wine in 1958. It was subsequently exported to Hong Kong. Recently, a compound anti-hangover beverage integrating mung bean polypeptide and ECG has been formulated [126,127,128,129]. ECG can also be processed into saccharide products. Its bitter taste can be improved by adding brown sugar. It can also be made into throat lozenges. The addition of menthol can impart a cool taste, thereby improving the overall flavor of ECG products [129]. A novel compound tea has been developed via co-fermentation. This formulation harmoniously combines tea and ECG aromas. ECG and green tea undergo co-fermentation to enhance the quality of the green tea [130]. Currently, the types of ECG teas include Huajuhong and Terminalia chebula tea, Huajuhong and Gardenia jasminoides tea, Huajuhong and Pu’er tea, as well as Huajuhong and Ampelopsis grossedentata tea. Adding ECG endows the teas with additional health-preserving effects [129]. Among these, fresh fruits, dried-fruit teas, Juhong wine, candied fruits, and throat lozenges are already commercially available, whereas the co-fermented compound teas and the anti-hangover beverage are still in the product-development or early-market stage.

ECG can be used not only in food but also in the manufacture of medicines. The functional components isolated from ECG can be further processed into cough-relieving and phlegm-resolving medications, namely traditional Chinese herbal products. Examples include Juhong TanKe granules, Juhong Pear paste, and Zhike Juhong oral liquid. Six types of formulations are covered, including pills, granules, extracts, syrups, tablets, and capsules. These products have been approved and are widely sold in the Chinese pharmaceutical market, while drug candidates targeting inflammation, oxidative stress, hyperglycemia, and hyperlipidemia are predominantly at the preclinical or laboratory stage.

Overall, ECG exhibits promising application potential across industrial, food, and pharmaceutical sectors. Notably, knowledge regarding gut microbiota-mediated metabolite transformation in humans remains limited. Although short-term rodent toxicity studies suggest the general safety of ECG, data on long-term toxicity, genotoxicity and reproductive toxicity are lacking. Further toxicological investigations are needed to define safety margins and support its functional development.

### 8.2. Product Development of ECG

**Figure 8:**
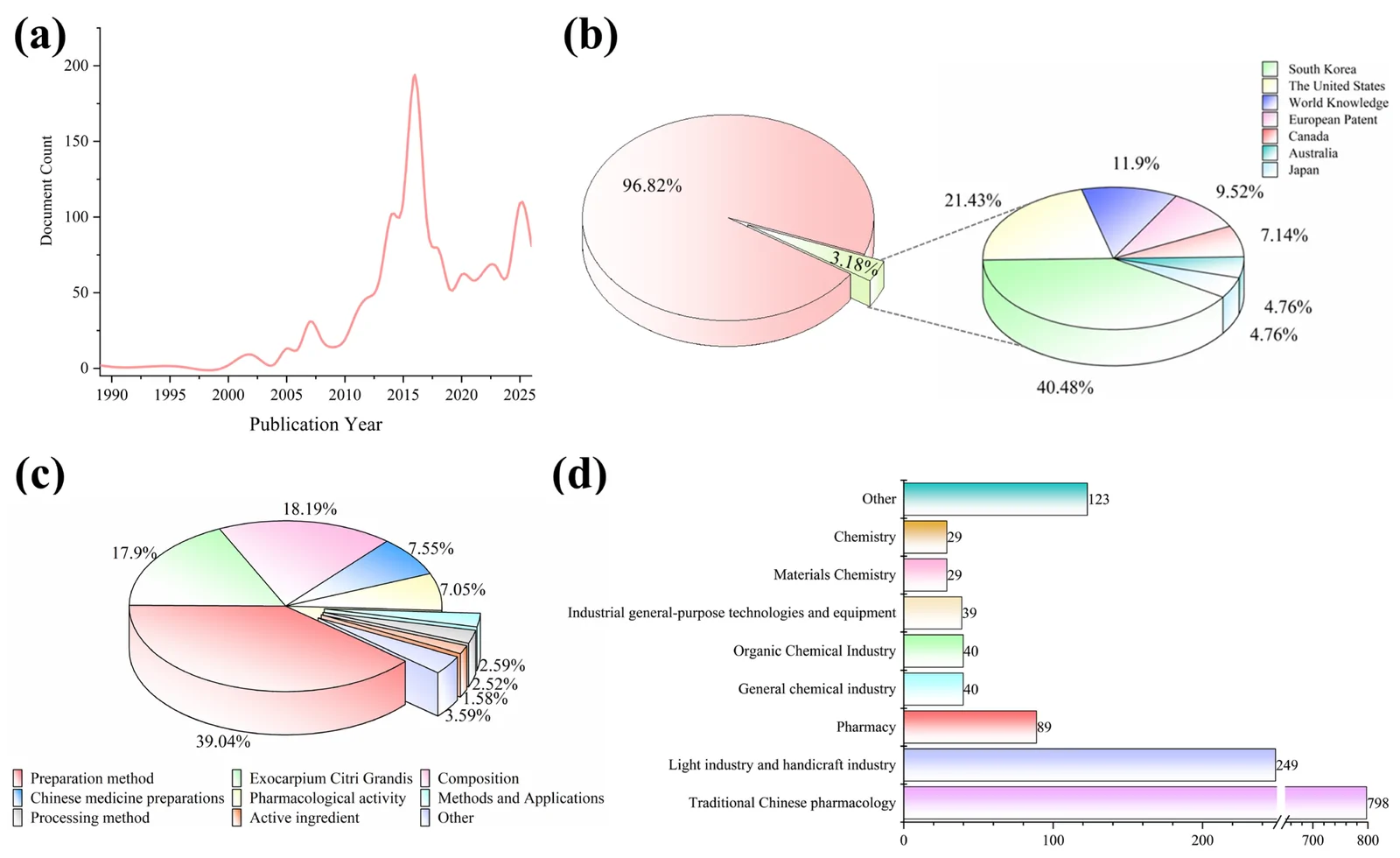
Analysis of 1414 patents related to ECG (publication trend (**a**); geographic distribution by nationalities of patent inventors (**b**); central product classification (**c**); distribution of technological categories (**d**)).

ECG possesses diverse biological activities. These activities endow ECG with great potential for application in product development. This work presents a detailed analysis of ECG-related patents (Figure 8). A total of 1414 patents associated with ECG published between 1989 and 2026 were screened. Patents related to ECG were scarce in the first decade. With an average annual publication volume of less than ten items, the number of patent publications first increased and then decreased after the start of the 21st century. It reached a peak in 2016 with 194 publications. Geographically, China is the main contributor. There are 1369 cumulative publications, accounting for 96.82%. Overseas patents were concentrated in South Korea and the United States, as well as at the European Patent Office. A total of 45 overseas invention patents were obtained. The analysis indicates that the internationalization process of technology exportation is still in the exploratory stage. The ECG patents currently focus primarily on applications, with few basic research patents. The main patent directions include traditional Chinese medicine preparations, preparation methods, pharmacological activities, and the preparation of ECG compositions, showing the characteristics of multidisciplinary integration.

## 9. Conclusions and Perspectives

This review has provided a comprehensive account of the botanical characteristics, traditional uses, phytochemistry, pharmacological activities, and applications of ECG across the food, pharmaceutical, and industrial sectors. It also provides a reference framework for quality control standardization, clinical translation, and product development. While considerable progress has been made, a number of unresolved issues deserve attention.

Regarding its botanical origin, ECG is derived from two botanical sources (CGT and CGO), yet their chemical and biological differences remain poorly defined. Moreover, recent studies have proposed expanding ECG’s medicinal parts to include immature fruits (Juhongtai), which exhibit superior pharmacological activity compared to mature pericarp [29]. However, the chemical basis underlying this superiority has not been systematically characterized. Regarding extraction technology, traditional extraction exhibits limitations, such as low efficiency and high solvent consumption. Developing green and efficient alternatives (e.g., deep eutectic solvent-assisted extraction, supercritical fluid extraction) has become a key direction. Current investigations into ECG’s chemical composition focus predominantly on flavonoids [131]. Volatile oils, polysaccharides, and coumarins have received insufficient attention. Future research ought to concentrate on their extraction, separation, purification, and structural identification. Notably, most of the 76 annotated flavonoids were tentatively characterized by mass spectrometry and lack NMR structural confirmation. Subsequent studies should focus on in-depth verification of these flavonoids. Research on the ADME profiles and toxicological properties of ECG remains limited, leaving its in vivo behavior poorly defined. In particular, long-term toxicity, genotoxicity, and reproductive toxicity data are lacking, and human pharmacokinetic evidence remains scarce. In terms of applications, the development of ECG for use in functional foods remains limited. Available products lack format diversity and are mostly restricted to traditional types such as herbal teas and preserves, which cannot satisfy the varied demands of consumers across different age groups and lifestyles. Further exploration of novel product formats (e.g., nanoemulsions, functional beverages, and co-fermented teas) is therefore warranted.

In conclusion, this review highlights the need for further research to characterize ECG’s medicinal properties and support its commercial translation. With improvements in standardization, mechanistic investigation and product development, ECG may evolve from a traditional medicinal-edible resource into a high-value functional ingredient with controllable quality and well-defined applications.

## Figures and Tables

**Table 1:** Aliases and origin information of ECG.

Dynasty	Aliases	Plant Origin	Medicinal Part	Refs
The Eastern Han Dynasty and earlier periods (before A.D. 220)	Juyou Jupi	*Citrus reticulata* Blanco *Citrus grandis* (L.) Osbeck *Poncirus trifoliata* (L.) Raf.	Pericarp	Ju Song Shen Nong Ben Cao Jing Jin Kui Yao Lue
The periods from the Jin dynasty, the Northern and Southern Dynasties to the Tang Dynasty (A.D. 266–907)	Jupi	*Citrus reticulata* Blanco *Citrus grandis* (L.) Osbeck	Exocarp	Ben Cao Jing Ji Zhu Lei Gong Pao Zhi Lun Shi Liao Ben Cao Ben Cao Shi Yi
The period from the Song Dynasty to the Yuan Dynasty (A.D. 960–1368)	Juhong	*Citrus reticulata* Blanco	Exocarp	Bo Zhai Bian Ben Cao Tu Jing Ben Cao Yan Yi
The Ming Dynasty (A.D. 1368–1644)	Juhong	*Citrus reticulata* Blanco *Citrus grandis* (L.) Osbeck *Poncirus trifoliata* (L.) Raf.	Exocarp	Ben Cao Pin Hui Jing Yao Ben Cao Gang Mu Ben Cao Hui Yan Ben Cao Meng Quan
The Qing Dynasty (A.D. 1636–1912)	Juhong Huazhou-chenpi Huazhou-juhong Huajuhong	*Citrus reticulata* Blanco *Citrus grandis* “Tomentosa” *Citrus grandis* (L.) Osbeck	Exocarp	Ben Cao Gang Mu Shi Yi Shi Yao Bian Wei Ling Nan Za Ji
The Republic of China period (1912–1949)	Juhong Lvmaohua Huazhoupi	*Citrus reticulata* Blanco *Citrus grandis* “Tomentosa” *Citrus grandis* (L.) Osbeck	Exocarp Whole immature fruit	Zeng Ding Wei Yao Tiao Bian Yao Wu Chu Chan Bian Ling Nan Cai Yao Lu
1949–1963	Juhong Youpi-juhong Maohua-juhong	*Citrus grandis* (L.) Osbeck *Citrus reticulata* Blanco *Citrus grandis* “Tomentosa”	Exocarp	Flora Of China Zhong Yao Zhi Guang Xi Zhong Yao Zhi
Chinese Pharmacopoeia (1963 Edition)	Juhong	*Citrus grandis* (L.) Osbeck *Citrus grandis* “Tomentosa” *Citrus reticulata* Blanco	Exocarp	Chinese Pharmacopoeia (1963 Edition)
Chinese Pharmacopoeia (1977 Edition)	Juhong	*Citrus grandis* (L.) Osbeck *Citrus grandis* “Tomentosa”	Immature exocarp	Chinese Pharmacopoeia (1977 Edition)
Chinese Pharmacopoeia (1985 ~2025 Edition)	Huajuhong	*Citrus grandis* (L.) Osbeck *Citrus grandis* “Tomentosa”	The dried outer pericarp of immature or nearly mature fruits	Chinese Pharmacopoeia (1985 ~2025 Edition)

**Table 2:** Traditional prescriptions containing ECG.

Type	Preparation Name	Herbal Compositions	Traditional Uses	Refs
Decoction	Huangqi Jishou Tang	*Exocarpium Citri Grandis*, *Ginseng Radix et Rhizoma*, *Chuanxiong Rhizoma*, *Paeoniae Radix Rubra*, etc.;	Treat carbuncles and cellulitis	Wai Ke Tu Shuo (Qing Dynasty, A.D. 1834)
	Feinongyang Tang	*Exocarpium Citri Grandis*, *Fritillariae Cirrhosae Bulbu*, *Platycodonis Radix*, *Descurainiae Semen*, *Lepidii Semen*, *Coicis Semen*, *Lonicerae Japonicae Flos*, *Forsythiae Fructus*, *Inulae Flos*, etc.;	Clear heat and toxins, descend qi to resolve phlegm, and drain pus	Lin Zheng Yi An Yi Fang (1981)
	Feiyan Tang	*Exocarpium Citri Grandis*, *Ephedrae Herba*, *Armeniacae Semen Amarum*, *Glycyrrhizae Radix et Rhizoma*, *gypsum Fibrosum*, *Arctii Fructus*, *Houttuyniae Herba*, *Fritillariae Cirrhosae Bulbus*, etc.;	Disperse wind-heat and relieve exterior syndrome, clear heat-toxin and remove toxins	Lin Zheng Yi An Yi Fang (1981)
	Tingli Wuzi Tang	*Exocarpium Citri Grandis*, *Descurainiae Semen*, *Lepidii Semen*, *Perillae Fructus*, *Armeniacae Semen Amarum*, *Raphani Semen*, *Fritillariae Cirrhosae Bulbus*, etc.;	Resolve phlegm to pacify wheezing, descend qi to suppress cough	Lin Zheng Yi An Yi Fang (1981)
	Dingchuan Tang No. 1	*Exocarpium Citri Grandis*, *Descurainiae Semen*, *Lepidii Semen*, *Perillae Fructus*, *Armeniacae Semen Amarum*, *Inulae Flos*, *Ephedrae Herba Belamcandae Rhizoma*, *Fritillariae Cirrhosae Bulbus*, etc.;	Pacify wheezing, dissipate phlegm, suppress cough, and redirect qi downward	Lin Zheng Yi An Yi Fang (1981)
	Dingchuan Tang	*Exocarpium Citri Grandis*, *Descurainiae Semen*, *Lepidii Semen*, *Perillae Fructus*, *Armeniacae Semen Amarum*, *Inulae Flos*, *Ephedrae Herba*, *Belamcandae Rhizoma*, *Fritillariae Cirrhosae Bulbus*, etc.;	Pacify wheezing, dissipate phlegm, suppress cough, and redirect qi downward	Lin Zheng Yi An Yi Fang (1981)
	Maxing Ersan Tang	*Exocarpium Citri Grandis*, *Ephedrae Herba*, *Armeniacae Semen Amarum*, *Pinelliae Rhizoma*, *Poria*, *Perillae Fructus*, *Raphani Semen*, *Glycyrrhizae Radix et Rhizoma*, etc.;	Dissipate phlegm-dampness, redirect qi to pacify wheezing, and fortify lung and spleen function	Jiao Shu De Fang (1998)
	Jiegeng Duhuo Tang	*Exocarpium Citri Grandis*, *Platycodonis Radix*, *Angelicae Pubescentis Radix*, etc.;	Treat gestational aphonia	Yi Fang Jian Yi (Qing Dynasty, A.D. 1883)
	Jiufei Shenghua Tang	*Exocarpium Citri Grandis*, *Persicae Semen*, *Chuanxiong Rhizoma*, *Angelicae Sinensis Radix*, *Glycyrrhizae Radix et Rhizoma*, *Fritillariae Cirrhosae Bulbus*, etc.;	Treat sepsis attacking the lung	Yi Fang Jian Yi (Qing Dynasty, A.D. 1883)
	Jiawei Yixin Tang	*Exocarpium Citri Grandis*, *Pinelliae Rhizoma*, *Poria*, *Glycyrrhizae Radix et Rhizoma*, *Polygalae Radix*, *Acori Tatarinowii Rhizoma*, *Codonopsis Radix*, etc.;	Tonify heart qi, warm the spleen, and resolve phlegm	Pu Fu Zhou Yi Liao Jing Yan
Pill	Fengxian Wan	*Exocarpium Citri Grandis*, *Agrimoniae Herba*, *Fritillariae Cirrhosae Bulbus*, *Angelicae Sinensis Radix*, *Bletillae Rhizoma*, *Glycyrrhizae Radix et Rhizoma*, etc.;	Treat hemoptysis, hematochezia, and metrorrhagia	Quan Guo Zhong Yao Chengyao Chu Fang Ji
	Feibing Tongxi Wan	*Exocarpium Citri Grandis*, *Fritillariae Cirrhosae Bulbus*, *Lilii Bulbus*, *Pinelliae Rhizoma*, *Glycyrrhizae Radix et Rhizoma*, *Armeniacae Semen Amarum*, *Mori Folium*, *Poria*, etc.;	Hydrate the lung to suppress dry cough, disperse phlegm, and pacify wheezing	Quan Guo Zhong Yao Cheng Yao Chu Fang Ji
	Juhong Wan	*Exocarpium Citri Grandis*, *Citri Reticulatae Pericarpium*, *Pinelliae Rhizoma*, *Poria*, *Glycyrrhizae Radix et Rhizoma*, *Platycodonis Radix*, *Armeniacae Semen Amarum*, *Perillae Fructus*, *Asteris Radix et Rhizoma*, etc.;	Facilitate lung purification, eliminate phlegm, and reduce cough	Bei Jing Shi Zhong Yao Cheng Fang Xuan Ji
	Juhong Huatan Wan	*Exocarpium Citri Grandis*, *Physalis Calyx Seu Fructus*, *Fritillariae Cirrhosae Bulbus*, *Armeniacae Semen Amarum*, *Schisandrae Chinensis Fructus*, *Glycyrrhizae Radix et Rhizoma*, etc.;	Consolidate the lung, dispel phlegm, alleviate cough and wheezing	Quan Guo Zhong Yao Cheng Yao Chu Fang Ji (Tianjin Fang)
	Shugan Jieyu Wan	*Exocarpium Citri Grandis*, *Magnoliae Officinalis Cortex*, *Aucklandiae Radix*, *Cyperi Rhizoma*, *Amomi Fructus*, *Pinelliae Rhizoma*, *Aquilariae Lignum Resinatum*, *Paeoniae Radix Alba*, etc.;	Strengthen the spleen and stomach, dispel stagnation, relieve depression, regulate qi, promote digestion, and alleviate pain	Quan Guo Zhong Yao Cheng Yao Chu Fang Ji (Shenyang Fang)
Tablet	Juhong Pian	*Exocarpium Citri Grandis*, *Citri Reticulatae Pericarpium*, *Pinelliae Rhizoma Praeparatum*, *Poria*, *Glycyrrhizae Radix et Rhizoma*, *Platycodonis Radix*, *Armeniacae Semen Amarum*, etc.;	Clear lung heat, resolve phlegm, and suppress cough	Bei Jing Shi Zhong Cheng Yao Gui Fan
Medicinal Qu	Jubei Banxia Qu	*Exocarpium Citri Grandis*, *Fritillariae Cirrhosae Bulbus*, *Pinelliae Rhizoma*, *Armeniacae Semen Amarum*, *Polygalae Radix*, *Platycodonis Radix*, *Glycyrrhizae Radix et Rhizoma*, etc.;	Ventilate the lung to dissipate phlegm, suppress cough, and pacify wheezing	Shang Hai Shi Yao Pin Biao Zhun
Paste	Lipi Hegan Huashi Gao	*Exocarpium Citri Grandis*, *Panacis Quinquefolii Radix*, *Scrophulariae Radix*, *Polyporus*, *Alismatis Rhizoma*, *Inulae Flos*, *Aurantii Fructus***,** etc.;	Modulate splenic transport to transform dampness	Ci Xi Guang Xu Yi Fang Xuan Yi

**Table 3:** The contents of amino acids in ECG [34].

Name	Content (mg/100 mL)		
	Fruit	Flower	Leaf
Essential amino acids			
L-threonine	1.73	5.72	1.33
Phenylalanine	1.47	4.80	0.54
Valine	0.90	3.94	0.38
L-lysine	0.33	2.09	0.76
DL-tryptophan	0.15	2.16	1.03
L-isoleucine	0.25	1.66	0.20
L-histidine	0.17	0.60	0.72
L-methionine	0.02	ND	0.02
Non-essential amino acids			
Proline	5.69	19.58	59.48
Aspartic acid	3.06	14.6	0.66
L-alanine	2.57	8.00	0.44
DL-serine	1.61	3.93	0.45
4-aminobutanoic acid	0.64	3.62	2.48
Arginine	0.62	2.70	0.08
Tyrosine	0.35	1.31	0.30
Glycine	0.24	1.13	0.07
Cystine	0.23	0.32	0.14
L-glutamic acid	0.07	1.13	0.24
DL-ornithine	0.11	0.08	ND
Taurine	0.62	ND	ND
Total amino acids	20.83	77.37	69.32

**Table 4:** The contents of minerals in ECG [36,37].

Name	Content (μg/kg)		
	Peel	Pulp	Fruit
K	145.8	167.1	145.8
Cu	75.27	40.28	61.93
Mn	46.66	56.93	82.73
Ca	20.45	22.53	52.37
P	24.20	18.64	19.72
Mg	13.36	11.43	11.67
Fe	0.3251	0.2971	1.466
Zn	0.1226	0.0984	0.1530
Na	0.0615	0.05560	0.1571
Total minerals	326.25	317.36	376.00

**Table 5:** Methods for extracting flavonoids from ECG.

Category	Extraction Methods	Extracting Agent	Extraction Process	Extraction Yield	Source or Part	Refs
Conventional extraction	Sorbent extraction	Petroleum ether, methanol	Petroleum ethe: Solid–liquid ratio: 1:100 (g/mL) Time: 2.5 h Methanol: Solid–liquid ratio: 1:100 (g/mL) Time: 4 h	Naringin: 11.98%	ECG	[46]
	Leaching method	Ethanol (80%, *v*/*v*)	Temp: 80 °C Solid–liquid ratio: 1:50 (g/mL)	Total flavonoids (TF): 3.19%	ECG	[47]
		Water	Solid–liquid ratio: 1:30 Temp: 70 °C Time: 3 h	Naringin: 5.61%	ECG	[48]
Auxiliary process technology	Ultrasonic-assisted ethanol extraction method	Ethanol (50.8%, *v*/*v*)	Power: 180 W Time: 50 min Solid–liquid ratio: 1:60.9 (g/mL) Temp: 57.5 °C	Naringin: 7.87%	ECG	[49]
		Ethanol (30%, *v*/*v*)	Solid–liquid ratio: 1:25 (g/mL) Time: 90 min	TF: 6.5%	ECG	[50]
	Ultrasonic-assisted aqueous two-phase system extraction method	Ethanol (55%, *v*/*v*), ammonium sulfate (0.3 g/mL)	Temp: 50 °C Time: 30 min Solid–liquid ratio: 1:20 (g/mL)	TF: 5.03%	ECG flowers	[51]
	Microwave-assisted extraction method	Ethanol (70%, *v*/*v*)	Solid–liquid ratio: 1:15 (g/mL) Time: 5 min	TF: 4.48%	ECG	[52]
		Ethanol (80%, *v*/*v*)	Power: 1200 W Solid–liquid ratio: 1:30 (g/mL) Temp: 90 °C Time: 15 min	TF: 3.10–3.35%	ECG	[53]
	Enzyme-assisted extraction method	Cellulase : Pectinase = 2:1	Enzyme dosage: 2% PH: 4.0 Temp: 50 °C Enzymolysis time: 45 min Ultrasonic time: 20 min Solid–liquid ratio: 1:25 (g/mL)	TF: 1.80%	ECG	[54]
Novel extraction methods	High-pressure difference continuous extraction method	-	Number of pulses: 250 Electric field strength: 7.8 kv/cm; Solid–liquid ratio: 1:70 (g/mL)	TF: 3.034%	ECG	[55]
	Deep eutectic solvent extraction method	Choline chloride–ethylene glycol	Moisture content: 43% Solid–liquid ratio: 1∶21 (g/mL) Temp: 60 °C	TF: 1.88–1.98%	ECG	[56]
	Ultrasonic-assisted natural deep eutectic solvent method	Choline chloride–glycerol–lactic acid	Solid–liquid ratio: 1:35 (g/mL) Moisture content: 30% Temp: 60 °C Time: 15 min	TF: 1.80%	ECG	[57]
	Subcritical fluid extraction technology	Water	Temp: 145.3–165.6 °C Flow rate: 2.25 mL/min	TF: 87.8–98.9%	Citrus unshiu peel	[58]

**Table 6:** Lists of methods reported in the literature for purification, identification and quantification of flavonoids from ECG.

Purification	Purifying Agent	Identification	Quantification	Refs
Macroporous resin column Chromatography	Ethanol	UPLC-DAD-MS/MS	UV, HPLC	[81]
Sephadex LH20 gel chromatography	30%, 85% ethanol	HPLC and HPLC-MS/MS	NaNO_2_-AlCl_3_-NaOH	[82]
High-speed Countercurrent chromatography	N-hexane-1-butanol–methanol-0.5% acetic acid (1:3:1:4, *v*/*v*)	ESI-MS	HPLC	[79]
AB-8 macroporous resin	Water and 50% ethanol	HPLC-MS	UV	[74]

**Table 7:** The flavonoids reported from ECG.

No.	Compounds	Molecular Formula	CAS	Identification Methods	Level of Confidence	Source	Refs
**Flavones** **(1** **–** **30)**							
1	rhoifolin	C_27_H_30_O_14_	17306-46-6	IR, NMR, HPLC-DAD-MS/MS	Level 1	ECG	[95]
2	vicenin-2	C_27_H_30_O_15_	23666-13-9	UFLC-Q-TOF-MS/MS	Level 2	ECG	[6]
3	diosmin	C_28_H_32_O_15_	520-27-4	UFLC-Q-TOF-MS/MS	Level 2	ECG	[6]
4	neodiosmin	C_28_H_32_O_15_	38665-01-9	UFLC-Q-TOF-MS/MS	Level 2	ECG	[6]
5	luteolin-6-C-glucoside	C_21_H_20_O_11_	4261-42-1	UFLC-Q-TOF-MS/MS	Level 2	ECG	[6]
6	isovitexin	C_21_H_20_O_10_	29702-25-8	LC-MS	Level 1	CGT	[96]
7	vitexin	C_21_H_20_O_10_	3681-93-4	LC-MS	Level 2	CGT	[96]
8	luteolin	C_15_H_10_O_6_	491-70-3	LC-MS	Level 2	CGT	[96]
9	luteolin-7-glucoside	C_21_H_20_O_11_	5373-11-5	LC-MS	Level 2	CGT	[96]
10	apiin	C_26_H_28_O_14_	26544-34-3	LC-MS	Level 2	CGT	[96]
11	chrysin	C_15_H_10_O_4_	480-40-0	LC-MS	Level 1	CGT	[96]
12	apigenin	C_15_H_10_O_5_	520-36-5	IR, NMR, HPLC-DAD-MS/MS	Level 1	CGT	[96]
13	lonicerin	C_27_H_30_O_15_	25694-72-8	LC-Q-TOF-MS/MS	Level 2	ECG	[97]
14	luteolin-7-rutinoside	C_27_H_30_O_15_	20633-84-5	LC-Q-TOF-MS/MS	Level 2	ECG	[97]
15	isorhoifolin	C_27_H_30_O_14_	552-57-8	UFLC–DAD–Q-TOF-MS/MS	Level 1	CGT	[87]
16	isosinensetin	C_20_H_20_O_7_	17290-70-9	HPLC	Level 2	ECG	[98]
17	sinensetin	C_20_H_20_O_7_	2306-27-6	HPLC	Level 2	ECG	[98]
18	trimethylapigenin	C_18_H_16_O_5_	5631-70-9	HPLC	Level 2	ECG	[98]
19	tangeretin	C_20_H_20_O_7_	481-53-8	UHPLC-Q-TOF/MS	Level 2	ECG	[99]
20	nobiletin	C_21_H_22_O_8_	478-01-3	UPLC-Q/TOF-MS	Level 2	ECG	[100]
21	5-hydroxy-3′,4′,6,7,8-pentamethoxyflavone	C_20_H_20_O_8_	/	UPLC-Q/TOF-MS	Level 2	ECG	[100]
22	5-hydroxy-3′,4′,3,6,7,8-hexamethoxyflavone	C_21_H_22_O_9_	/	UPLC-Q/TOF-MS	Level 2	ECG	[100]
23	lucenin-2,4′-methylether	C_28_H_32_O_16_	/	UFLC-Q-TOF-MS/MS	Level 2	ECG	[6]
24	vitexin--2″-*O*-xyloside	C_26_H_28_O_14_	/	LC-Q-TOF-MS/MS	Level 2	ECG	[97]
25	isovitexin-7-*O*-xylocoside-2″-O-arabinoside	C_31_H_36_O_18_	/	LC-Q-TOF-MS/MS	Level 2	ECG	[97]
26	diosmetin-7-*O*-(6″-*O*-acetyl)-neohesperidoside	C_30_H_34_O_17_	/	LC-Q-TOF-MS/MS	Level 2	ECG	[97]
27	apigenin-8-C-glucoside-*O*-arabinoside	C_26_H_28_O_14_	/	RRLC-ESI-Q-TOF/MS	Level 2	CGT	[84]
28	apigenin-6-C-glucoside-*O*-arabinoside	C_26_H_28_O_14_	/	RRLC-ESI-Q-TOF/MS	Level 2	CGT	[84]
29	apigenin-8-C-glucoside-*O*-rhamnoside	C_27_H_30_O_14_	/	RRLC-ESI-Q-TOF/MS	Level 2	CGT	[84]
30	apigenin-6-C-glucoside-*O*-rhamnoside	C_27_H_30_O_14_	/	RRLC-ESI-Q-TOF/MS	Level 2	ECG	[84]
**Flavanones** **(31** **–** **46)**							
31	naringin	C_27_H_32_O_14_	10236-47-2	IR, NMR, HPLC-DAD-MS/MS	Level 1	ECG	[95]
32	naringenin	C_15_H_12_O_5_	480-41-1	IR, NMR, HPLC-DAD-MS/MS	Level 1	ECG	[95]
33	melitidin	C_33_H_40_O_18_	1162664-58-5	IR, NMR, HPLC-DAD-MS/MS	Level 1	ECG	[95]
34	poncirin	C_28_H_34_O_14_	14941-08-3	IR, NMR, HPLC-DAD-MS/MS	Level 1	ECG	[95]
35	neohesperidin	C_28_H_34_O_15_	13241-33-3	IR, NMR, HPLC-DAD-MS/MS	Level 1	ECG	[95]
36	neoeriocitrin	C_27_H_32_O_15_	13241-32-2	IR, NMR, HPLC-DAD-MS/MS	Level 1	ECG	[95]
37	narirutin	C_27_H_32_O_14_	14259-46-2	UFLC-Q-TOF-MS/MS	Level 2	ECG	[6]
38	narirutin-4′-*O*-glucoside	C_33_H_42_O_19_	17257-22-6	UFLC-Q-TOF-MS/MS	Level 2	ECG	[6]
39	naringin-4′-*O*-glucoside	C_33_H_42_O_19_	17257-21-5	UFLC-Q-TOF-MS/MS	Level 2	ECG	[6]
40	eriocitrin	C_27_H_32_O_15_	13463-28-0	UFLC-Q-TOF-MS/MS	Level 2	ECG	[6]
41	hesperidin	C_28_H_34_O_15_	520-26-3	UFLC-Q-TOF-MS/MS	Level 2	ECG	[6]
42	hesperetin	C_16_H_14_O_6_	520-33-2	LC-MS	Level 2	CGT	[96]
43	pinocembrin	C_15_H_12_O_4_	480-39-7	LC-MS	Level 2	CGT	[96]
44	naringenin-7-*O*-beta-D-glucoside	C_21_H_22_O_10_	529-55-5	LC-MS	Level 2	CGT	[96]
45	eriodictyol	C_15_H_12_O_6_	552-58-9	LC-MS	Level 2	CGT	[96]
46	didymin	C_28_H_34_O_14_	14259-47-3	HPLC	Level 2	CGT	[98]
**Isoflavones** **(47** **–** **51)**							
47	glycitin	C_22_H_22_O_10_	40246-10-4	LC-MS	Level 2	CGT	[96]
48	daidzin	C_21_H_20_O_9_	552-66-9	LC-MS	Level 2	CGT	[96]
49	genistein	C_15_H_10_O_5_	446-72-0	LC-MS	Level 2	CGT	[96]
50	genistin	C_21_H_20_O_10_	529-59-9	LC-MS	Level 2	CGT	[96]
51	glycitein	C_16_H_12_O_5_	40957-83-3	LC-MS	Level 2	CGT	[96]
**Flav** **o** **nols** **(52** **–** **61)**							
52	kaempferol	C_15_H_10_O_6_	520-18-3	UFLC-Q-TOF-MS/MS	Level 1	ECG	[6]
53	rutin	C_27_H_30_O_16_	153-18-4	LC-MS	Level 2	CGT	[96]
54	quercetin	C_15_H_10_O_7_	117-39-5	LC-MS	Level 1	CGT	[96]
55	quercetin-3-*O*-glucoside	C_21_H_20_O_12_	482-35-9	LC-MS	Level 2	CGT	[96]
56	kaempferol-3-*O*-rutinoside	C_27_H_30_O_15_	6130-58-1	LC-MS	Level 2	CGT	[96]
57	isorhamnetin	C_16_H_12_O_7_	480-19-3	LC-MS	Level 2	CGT	[96]
58	isorhamnetin-3-*O*-glucoside	C_22_H_22_O_12_	6743-92-6	LC-Q-TOF-MS/MS	Level 2	ECG	[97]
59	limocitrin-3-rhamnoside	C_23_H_24_O_12_	/	LC-Q-TOF-MS/MS	Level 2	ECG	[97]
60	troxerutin	C_33_H_42_O_19_	7085-55-4	LC-Q-TOF-MS/MS	Level 2	ECG	[97]
61	kaempferol-3-(2′-rhamnosylrutinoside)	C_33_H_40_O_19_	/	LC-Q-TOF-MS/MS	Level 2	ECG	[97]
**Flavanonols** **(62, 63)**							
62	taxifolin	C_15_H_12_O_7_	480-18-2	LC-MS	Level 2	CGT	[96]
63	dihydrokaempferol	C_15_H_12_O_6_	480-20-6	UHPLC-Q-Exactive	Level 2	ECG	[6]
**Anthocyanidins** **(64** **–** **68)**							
64	pelargonidin	C_15_H_11_O_5_	7690-51-9	LC-MS	Level 2	CGT	[96]
65	peonidin-3-glucoside	C_22_H_23_ClO_11_	6906-39-4	LC-MS	Level 2	CGT	[96]
66	delphinidin-3-glucoside	C_21_H_21_ClO_12_	26984-07-6	LC-MS	Level 2	CGT	[96]
67	cyanidin-3-galactoside	C_21_H_21_ClO_11_	27214-71-7	LC-MS	Level 2	CGT	[96]
68	delphinidin-3-*O*-*β*-D-sambubioside	C_26_H_29_ClO_16_	53158-73-9	LC-MS	Level 2	CGT	[96]
**Flavan-3-ols** **(69** **–** **71)**							
69	catechin	C_15_H_14_O_6_	154-23-4	LC-MS	Level 2	CGT	[96]
70	epicatechin	C_15_H_14_O_6_	490-46-0	LC-MS	Level 2	CGT	[96]
71	(-)-epigallocatechin	C_15_H_14_O_7_	970-74-1	LC-MS	Level 2	CGT	[96]
**Flavan-3,4-diols** **(72, 73)**							
72	loquatoside	C_20_H_22_O_11_	74046-15-4	LC-Q-TOF-MS/MS	Level 2	ECG	[97]
73	leucopelargonidin	C_15_H_14_O_6_	520-17-2	LC-MS	Level 2	CGT	[96]
**Dihydrochalcones** **(74, 75)**							
74	phloretin-3′,5′-di-C-glucoside	C_27_H_34_O_15_	357401-40-2	LC-Q-TOF-MS/MS	Level 2	ECG	[97]
75	hesperetin dihydrochalcone-4-*β*-D-glucoside	C_23_H_30_O_10_	520-33-2	LC-Q-TOF-MS/MS	Level 2	ECG	[97]
**Chalcones** **(76)**							
76	naringenin chalcone	C_15_H_12_O_5_	73692-50-9	UHPLC-Q-Exactive	Level 2	ECG	[6]

Level 1: structure established by isolation and NMR or by co-elution with an authentic standard; Level 2: structure assigned from MS/MS fragments, spectral library or literature comparison, without a reference standard.

**Table 8:** Summary of activities of the extract/compounds from ECG.

Health Benefits	Extract or Compound	Dosage and Administration	Study Design	Experimental Model	Modeling Methods	Main Findings	Refs
hypoglycemic and hypolipidemic effects	TFECG and naringin	200, 100 mg/kg; 28 days (i.g.)	in vivo	SD mice	HFD combined with twice STZ intraperitoneal-injection-induced diabetic model	↓ TC, TG, LDL-C, liver index, kidney index; ↑ HDL-C, insulin level	[74]
hypoglycemic and hypolipidemic effects	the extract of ECG	180, 360, and 720 mg/kg (i.g.)	in vivo	SD rats	HFD-induced hyperlipidemic model	↓ TG, TC, LDL-C; exert hypolipidemic activity through the PXRCYP3A4/FXR-LXR*α* mechanism	[107]
hypoglycemic and hypolipidemic effects	the extract of ECG	2.5, 5, and 10 g/kg; 6 weeks (i.g.)	in vivo	C57BL/6J mice	HFD-induced NAFLD mouse model	↓ SREBP1, HEPCIDIN, lipid accumulation; ↑ STEAP3, Fpn1; jointly improve abnormal lipid metabolism	[108]
anti-inflammatory effects	TFECG	40, 80, and 160 mg/kg (i.g.)	in vivo	Balb/c mice	single intratracheal instillation of PM_2.5_ suspension-induced acute lung injury model	↓ WBC, LYMPH, NEUT, MONO, TNF-*α*, IL-1*β*, IL-6, IL-18	[42]
anti-inflammatory effects	the extract of ECG	25, 50, and 100 μg/mL	in vitro	RAW264.7 macrophages	LPS-induced inflammatory model	↓ IL-1*β*, IL-6, TNF-*α*, NO, TLR4, MyD88, NF-κB p65 (and p-p65), p-IκB*α*, COX2, ICAM1, iNOS; inhibit the TLR4/MyD88/NF-κB signaling pathway; ↑ IκB*α*	[110]
antioxidant effects	TFECG	40, 80, and 160 mg/kg (i.g.)	in vivo	Balb/c mice	single intratracheal instillation of PM_2.5_ suspension-induced acute lung injury model	↓ TP, MDA, NO, LDH, iNOS; ↑ SOD, GSH-Px, GSH/GS ratio	[42]
antioxidant effects	TFECG	in vitro: 0.24–1.2 mg/mL; in vivo: 50, 100, and 200 mg/kg; 28 days (i.g.)	in vivo	in vitro chemical assay; male SD rats	in vitro: DPPH-scavenging, reducing-power and total-antioxidant-capacity assays; in vivo: HFD -induced hyperlipemia model	↓ MDA, TC, LDL-C; ↑ SOD	[72]
antitussive, expectorant and antiasthmatic effects	the extract of ECG	mice: 20, 40, and 80 mg/kg; guinea pig: 12, 24, and 48 mg/kg; rat: 14, 28, and 56 mg/kg; 7 days (i.g.)	in vivo	mice, rats, guinea pigs	ammonia-induced cough model; citric acid-induced cough model; phenol-red-excretion mouse expectorant model; capillary-tube sputum-secretion model; histamine-acetylcholine-induced asthma model	↓ cough frequency; ↑ ammonia spray time, cough latent period, phenol red excretion, sputum volume, asthma latent period	[41]
antitussive, expectorant and antiasthmatic effects	crude drugs of ECG (water, 50% ethanol, 70% ethanol and 90% ethanol)	aqueous extract (1005 mg/kg); 50% ethanolic extract (568 mg/kg); 70% ethanolic extract (247, 493, and 986 mg/kg); 90% ethanolic extract (501 mg/kg); 5 days (i.g.)	in vivo	NIH mice	ammonia-induced cough model; tracheal phenol-red secretion expectorant model; xylene-induced ear-edema anti-inflammatory model	↓ cough frequency; ↑ cough latent period, tracheal phenol red secretion; the 70% ethanolic extract exerts the strongest, dose-dependent antitussive and expectorant effects	[109]
hepatoprotective, anti-alcoholic, lipid-lowering effects	ECG aqueous extract	in vivo: zebrafish: 20, 40, and 80 µg/mL; mice: 1.67, 3.34, and 6.68 g/kg/d, 3 weeks (i.g.); in vitro: 2.5%, 5%, and 10% ECG-containing serum, 6 g/kg/d	in vivo and in vitro	zebrafish, C57BL/6 mice, AML-12 hepatocytes, RAW264.7 macrophages, RAGE-overexpression cell model	alcohol-induced alcoholic liver disease model; Lieber–DeCarli alcohol liquid-diet-induced alcoholic liver disease model; alcohol-stimulated AML-12 and RAW264.7 cell injury model; RAGE overexpression cell intervention model	↓ ALT, AST, TG, TC, SREBP1, TNF-*α*, IL-6, IL-1*β*, CCL2, iNOS, Hepcidin, MDA, 4-HNE; ↑ GSH, SOD, GPx; ameliorate alcohol-induced hepatic steatosis and liver injury by inhibiting RAGE/p-NF-κB signaling	[114]
lung injury protection/anti-inflammation and antioxidant effects	the extract of ECG	Mice: 25, 50 mg/kg; 7 days (i.g.); RAW264.7 cell: 25, 50, and 100 μg/mL	in vivo and in vitro	C57BL/6 mice, RAW264.7 macrophages	intraperitoneal injection of LPS (5 mg/kg) -induced acute lung injury model; LPS-stimulated inflammatory model	↓ IL-1*β*, IL-6, TNF-*α*, ROS, MDA, iron, TLR4, MyD88, NF-κB, COX2, iNOS, JAK, STAT, Txnip, NLRP3, ASC, Caspase-1, Keap1; ↑ SOD, GSH, Trx-1, Nrf2, HO-1, NQO1, GPX4, PCBP1, SLC40A1; inhibit TLR4/MyD88/NF-κB, JAK/STAT, TXNIP/NLRP3	[115]
hepatoprotection and gut microbiota regulation	the extract of ECG	296.4 mg/kg; 30 days (i.g.)	in vivo	ICR mice	polystyrene microplastic (PS)-induced hepatic-intestinal injury mouse model by continuous oral exposure to PS	↓ ALT, AST, MDA, TLR4, NF-κB, NLRP3, and inflammatory cell infiltration in the liver and colon; ↑ colon length, liver index, colonic mucus secretion and number of goblet cells, T-AOC, SOD, CAT, Nrf2, HO-1, Occludin, Claudins, ZO-1	[116]
anti-asthmatic/antitussive effects	naringin	18.4 mg/kg; 6 days (i.g.)	in vivo	hartley strain guinea pigs	ovalbumin-sensitized cough-variant asthma (CVA) model by intraperitoneal sensitization and aerosol ovalbumin challenge	↓ enhanced cough and airway hyperresponsiveness, IL-4, IL-5, and IL-13	[117]
anti-obesity	the extract of ECG	50, 100 mg/kg; 90 days (i.g.)	in vivo	female C57BL/6J mice	bilateral ovariectomy-induced postmenopausal obesity with hepatic lipid-metabolic disorder model	↓ body weight, TG, TC, LDL-C, adipocyte hypertrophy, Acc, Fas, SREBP1C, TNF-α, IL-6; ↑ HDL-C, oxygen consumptionVO2, carbon dioxide output VCO2, heat production	[118]

↓: indicates reduced biomarker levels, suppressed protein expression, or inhibited signaling pathway activity, while the upward arrow; ↑: denotes elevated biomarker levels, increased protein expression, or activated pathway responses.

## Data Availability

No new data were created or analyzed in this study. Data sharing is not applicable to this article.

## Abbreviations

The following abbreviations are used in this manuscript:

ECG	Exocarpium Citri Grandis
CGT	*Citrus grandis* “Tomentosa”
CGO	*Citrus grandis* (L.) Osbeck
TCM	Traditional Chinese Medicine
COVID-19	Coronavirus Disease 2019
SFE	Supercritical Fluid Extraction
CE	Conventional Solvent Extraction
SC-CO2	Supercritical Carbon Dioxide
DPPH	2,2-Diphenyl-1-picrylhydrazyl
ABTS	2,2′-Azino-bis (3-ethylbenzothiazoline-6-sulfonic acid)
MIT	Molecular Imprinting Technology
HPCCC	High-Performance Counter-Current Chromatography
HPLC	High-Performance Liquid Chromatography
RP-HPLC	Reversed-phase High-Performance Liquid Chromatography
TLC	Thin-Layer Chromatography
LC/MS	Liquid Chromatography/Mass Spectrometry
NMR	Nuclear Magnetic Resonance
UFLC-DAD-Q-TOF-MS/MS	Ultra-Fast Liquid Chromatography, Diode Array Detection, Quadrupole Time-Of-Flight Mass Spectrometry
AQP	Aquaporin
PCO	Protein Carbonyls
CAT	Catalase
TFECG	Total Flavonoids of Exocarpium Citri Grandis
TFs	Total Flavonoids
TG	Triglyceride
TC	Total Cholesterol
LDL	Low-Density Lipoprotein
NAFLD	Non-Alcoholic Fatty Liver Disease
SREBP1	Sterol Regulatory Element-Binding Protein 1
STEAP3	Six-Transmembrane Epithelial Antigen of the Prostate 3
Fpn1	Ferroportin 1
TLRs	Toll-Like Receptors
WBCs	White Blood Cells
LYMPHs	Lymphocytes
NEUTs	Neutrophils
MONOs	Monocytes
LTB4	Leukotriene B4
LXA4	Lipoxin A4
MPO	Myeloperoxidase
LDH	Lactate Dehydrogenase
iNOS	Inducible Nitric Oxide Synthase
TP	Total Protein
GSH-Px	Glutathione Peroxidase
GSH	Glutathione
GSSG	Oxidized Glutathione
SOD	Superoxide Dismutase
ROS	Reactive Oxygen Species
LOOH	Lipid Hydroperoxides
NO_2_^−^	Nitrite
MDA	Malondialdehyde
